# Integrated ICD-based subtyping and prognostic model reveal KCNN4 as a novel therapeutic target in NSCLC

**DOI:** 10.1007/s12672-026-04651-8

**Published:** 2026-02-25

**Authors:** Zhengzheng Yang, Qianru Yang, Shujiao Li, Ruiyang Han, Haiqiu He, Runze Zhang, Haiming Li, Yudong Li, Peiyu Cheng, Xiawei Liu

**Affiliations:** 1 Beijing Hospital of Traditional Chinese Medicine, Capital Medical University, No. 23, Houjie, Meishuguan, Dongcheng District, Beijing, China; 2 Dongzhimen Hospital, Beijing University of Chinese Medicine, Beijing, China; 3 China Academy of Chinese Medical Sciences Eye Hospital, Beijing, China; 4 Beijing Hospital of Traditional Chinese Medicine, Beijing University of Chinese Medicine, Beijing, China; 5 Beijing University of Chinese Medicine Third Affiliated Hospital, No. 13, Anyuanli, Xiaoguan Xiejie, Anzhenmen, Chaoyang District, Beijing, China

**Keywords:** Non-small cell lung cancer, Immunogenic cell death, Tumor microenvironment, Prognostic model, Molecular dynamics simulations

## Abstract

**Backgrounds:**

Non-small cell lung cancer (NSCLC), the most common type of lung cancer, stands as a leading cause of cancer-related mortality worldwide. Immunogenic cell death (ICD )enhances cancer therapy efficacy by inducing immune responses and reshaping the TIME. While ICD increases cytotoxic T lymphocyte infiltration and reduces immunosuppressive elements, the specific TIME subtypes associated with ICD in lung adenocarcinoma (LUAD) remain undefined.

**Methods:**

Publicly available datasets from The Cancer Genome Atlas (TCGA) and the Gene Expression Omnibus (GEO) were utilized in this study to identify differentially expressed genes related to ICD. We applied consensus molecular clustering to integrate these genes with clinical phenotypes, revealing distinct NSCLC subtypes with varying prognostic outcomes. The tumor immune microenvironments of these clusters were characterized using the ‘estimate’ R package ‘and ‘CIBERSORT’.A prognostic model was established utilizing LASSO Cox regression and validated across independent cohorts. Functional validation involved RNA interference targeting the KCNN4 gene in PC-9 lung cancer cells, including quantitative PCR, cell proliferation assays, wound healing assays, and Transwell invasion assays. Additionally, molecular docking and molecular dynamics simulations were performed to identify and validate small-molecule drugs targeting KCNN4.

**Results:**

Unsupervised clustering of ICD-related gene expressions delineated three novel NSCLC subtypes. Cluster 1, characterized by younger patients, exhibited enhanced immune activity with significant infiltration of activated CD4^+^ and CD8^+^ T cells, correlating with a favorable prognosis and responsiveness to immunotherapy. Cluster 2, predominantly female, displayed suppressed immune responses with reduced effector memory T cells and γδ T cells, associated with poorer outcomes. Cluster 3, linked to varying cancer stages, showed moderate immune activity with lower immune cell infiltration and higher tumor purity, indicating an intermediate prognosis. A comprehensive prognostic model combined the expression levels of five key ICD-related genes (CSF2RB, CD3D, ADA2, KCNN4, and AREG) with critical clinical factors. Targeted silencing of KCNN4 in PC-9 cells significantly reduced tumor aggressiveness, supporting its role as a therapeutic target. Molecular docking identified four promising small-molecule drugs, with ZINC000000001547 (Hydroxystilbamidine) showing stable interactions in molecular dynamics simulations.

**Conclusions:**

This study identifies three distinct ICD-related TIME subtypes in NSCLC with significant translational potential: Cluster 1 patients are ideal candidates for immune checkpoint inhibitors; Cluster 2 may benefit from combination therapies to overcome resistance; and Cluster 3 requires aggressive multimodal treatments. Additionally, the predictive model enhances risk stratification, improving patient outcomes in NSCLC.

**Supplementary Information:**

The online version contains supplementary material available at 10.1007/s12672-026-04651-8.

## Introduction

Recent estimates reveal an overwhelming global impact, with around 20 million new cancer diagnoses and nearly9.7 million cancer-related fatalities recorded in 2022 alone [1]. Lung cancer continues to be the foremost cause of death due to cancer, claiming around 1.8 million lives(18.7%), with colorectal, liver, female breast and stomach cancers following in prevalence [1]. Non-small cell lung cancer (NSCLC) constitutes the majority of these cases, encompassing various histological types. Among these, lung adenocarcinoma (LUAD) is the most common subtype, making up nearly 60% of cases, with its incidence continuing to rise. Despite progress in molecular biology and personalized treatments, the 5-year overall survival (OS) rate for LUAD patients remains a low 14.6% [2].

Previous studies have provided important insights into the clinical characteristics and therapeutic strategies of NSCLC. For example, Rice et al. highlighted the current status and future perspectives of immunotherapy in NSCLC [3]; Qian et al. demonstrated that the miR-451/ETV4/MMP13 signaling axis promotes epithelial–mesenchymal transition and contributes to tumor progression [4]; and Cai et al. reported that the EGFR inhibitor CL-387,785 effectively suppresses the progression of lung adenocarcinoma [5] These findings collectively underscore the urgent need for novel prognostic biomarkers and therapeutic targets in NSCLC.

Immunogenic cell death (ICD) represents a unique category of cell demise elicited by various anticancer therapies, including radiotherapy and chemotherapeutic agents. In immunocompetent hosts, ICD facilitates the immune system’s engagement against cancer by promoting the exposure and production of multiple damage-associated molecular patterns, which enhance the immunogenicity of dying tumor cells through the recruitment and activation of antigen-presenting cells [6]. ICD is characterized by the externalization of calreticulin (ecto-CRT), ATP secretion, and the release of high-mobility group box 1 protein (HMGB1). During ICD, ecto-CRT acts as an “eat me” signal, ATP functions as a “find me” signal, and HMGB1 serves as a potent cytokine, attracting various immune cells [7].

Enhancing the immunogenicity of tumor cells by inducing ICD is pivotal for augmenting the efficacy of cancer immunotherapy. It has been reported that cEMSY, an ICD-related circular RNA, elicits immunogenic cell death in lung adenocarcinoma cells in vitro and in vivo, while acting synergistically with PD-1 inhibition to amplify antitumor immune responses [8]. Shuai Zhang et al. discovered through in vitro and in vivo studies that ischemia-reperfusion (I/R) injury combined with the frontline chemotherapeutic agent cisplatin robustly induces the exposure of CRT, secretion of ATP, release of HMGB1, and phosphorylation of eIF2α in Lewis lung carcinoma (LLC) and A549 cells. They found that I/R significantly amplifies the antitumor effects of cisplatin and mitomycin C, thereby counteracting the immunosuppressive effects induced by cisplatin, and demonstrating that combining I/R with non-immunogenic chemotherapy represents a promising therapeutic strategy [9]. Furthermore, Peng Liu et al. demonstrated that administering cisplatin together with high-dose crizotinib can induce immunogenic cell death in NSCLC cells. This treatment combination results in the upregulation of PD-1 and PD-L1 expression within the tumor, thereby enhancing the effectiveness of immunotherapy [10]. Clinical studies have further validated the efficacy of inducing ICD. For example, a pivotal phase 3 randomized, open-label trial conducted by Ticiana Leal et al. included 276 patients with metastatic NSCLC. These patients were divided into two groups: one receiving TTFields therapy combined with standard systemic therapy or docetaxel, and a control group receiving only standard systemic therapy. The group treated with TTFields and standard therapy demonstrated significantly longer overall survival compared to the group receiving standard therapy alone [11].

ICD is pivotal in oncology therapy, primarily by eliciting an immune response and reconfiguring the tumor immune microenvironment (TIME). ICD not only results in the direct elimination of tumor cells but also modulates the TIME by increasing the infiltration of cytotoxic T lymphocytes and diminishing immunosuppressive elements, thereby enhancing antitumor immunity [12, 13]. Several studies have delineated the tumor microenvironmental states of lung squamous cell carcinoma (LUSC) and LUAD across distinct immune-infiltration patterns and profiled the genomic and clinical correlates of discrete immune phenotypes [14, 15]; however, the relationship of these phenotypes to ICD has not been elucidated. Although previous studies have classified NSCLC based on immune markers (e.g., tumor-infiltrating lymphocytes or PD-L1 expression), these approaches capture only part of the tumor–immune landscape. In contrast, our ICD-related classification integrates immunogenic cell death signatures, thereby providing a more comprehensive and clinically relevant framework.In this study, we integrated transcriptomic data from TCGA and GEO to identify ICD-related genes and classified NSCLC into three subtypes with distinct clinical, immune, and prognostic characteristics. Cluster 1 featured enhanced immune activity with significant infiltration of activated CD4^+^ and CD8^+^ T cells, correlating with younger patients and a favorable prognosis. Cluster 2, predominantly female, exhibited suppressed immune responses, characterized by reduced effector memory T cells and γδ T cells, associated with the poorest outcomes. Cluster 3 showed moderate immune activity with lower immune cell infiltration, including CD4^+^ and CD8^+^ T cells, indicating intermediate prognosis and higher tumor purity. We validated KCNN4, a key prognostic gene, via RNA interference in PC-9 cells, showing reduced tumor aggressiveness. Additionally, we screened small molecules targeting KCNN4, identifying four candidates. Molecular dynamics simulations confirmed stable interactions of KCNN4 with ZINC000000001547 (Hydroxystilbamidine), supporting its potential as a therapeutic target in NSCLC.

## Materials and methods

### Data sources and preprocessing

All the data utilized in this study are publicly accessible, primarily sourced from TCGA (Access CCG Data - NCI (cancer.gov)) and the GEO (GSE81089). The whole-genome expression profiles of lung cancer, in “transcripts per kilobase per million (TPM)” format, along with clinical annotations, were downloaded using the R package ‘TCGA biolinks’ from the TCGA database and ‘GEO query’ from the GEO database. FASTQ files underwent QC (FastQC), adapter/low-quality trimming, and two-pass alignment to GRCh38 (GENCODE v38) with STAR; gene-level counts were generated with featureCounts (gencode.v38.gtf), converted to TPM, and log₂(TPM + 1)-transformed for analysis. Gene identifiers were harmonized with biomaRt/org.Hs.eg.db, after which matrices were restricted to the intersection gene set. To curb technical noise, genes with TPM < 1 in ≥ 80% of samples per dataset were filtered out. Before integration, each dataset was mean-centered per gene on the log₂(TPM + 1) scale.Clinical annotation. The analytic cohort comprised TCGA-LUAD primary solid tumor (01A) and solid tissue normal (11A) specimens (n = 585; 522 tumors, 63 normals), together with GSE81089 RNA-seq samples (199 tumors, 19 controls).Where available, clinical and biospecimen fields encompassed age at diagnosis, sex, AJCC pathologic stage with T/N/M components, histologic grade, tumor status and vital status, days to event (death or recurrence) or last follow-up, prior malignancy, prior systemic therapy, adjuvant therapy, radiation therapy, and selected procedural variables (e.g., surgical resection status). Batch effects were adjusted using the R package “sva”. The 34 ICD-related genes were obtained from the study of Abhishek D. Garg [16].

### Differentially expressed genes associated with lung cancer

The “limma (version 3.50.0)” package in R was used to detect DEGs in the GSE81089 dataset, comparing normal and tumor tissues, with thresholds set at |log2FC|>0.5 and an adjusted *p* < 0.05. These DEGs were included in the subsequent research. ICD-related DEGs were derived from the intersection of the identified DEGs and the set of genes related to ICD.

### GO and KEGG pathway enrichment analysis

Gene Ontology (GO) enrichment analysis encompasses Biological Process (BP), Molecular Function (MF), and Cellular Component (CC) analyses. The Kyoto Encyclopedia of Genes and Genomes (KEGG) is a bioinformatics resource used to identify significantly altered metabolic pathways within the gene list. The R package “cluster Profiler (version 4.2.2)” was utilized to conduct GO and KEGG enrichment analyses (*p* < 0.05) on the genes within the protein-protein interaction (PPI) network.

### Consensus molecular clustering based on ICD-related genes and clinical phenotypes

Based on the expression levels ofICD -related molecules, a consensus clustering algorithm was used to classify cohorts of TCGA-LUAD and GSE81089 into novel ICD phenotypes in LUAD through the R package “Consensus Cluster Plus”. We evaluated k = 2–6 under the following settings: distance = “pearson” (i.e., 1 − Pearson correlation), clusterAlg = “hc” with inner/final linkage = “average”, reps = 1000, pItem = 0.8, pFeature = 1.0, seed = 202,401, and plot = “png”. For each k, we summarized stability using the consensus matrix, its empirical cumulative distribution function (CDF), and ΔCDF (area under CDF). We additionally computed the proportion of ambiguous clustering (PAC) defined as CDF(0.9) − CDF(0.1), the cluster-level consensus (mean pairwise consensus within each cluster), and the item-level consensus (mean consensus of each sample with members of its assigned cluster).Selection of k. The optimal solution was chosen as the smallest k at which ΔCDF plateaued, PAC was minimized, all cluster-level consensus scores were ≥ 0.80, and the median item-level consensus was ≥ 0.60 without yielding any cluster < 5% of the cohort. Optimal clusters were identified using the cumulative distribution function (CDF) showing the slowest rate of decline. Differences in clinical phenotypes, including age, gender, smoking history, relapse situation, and pathologic stage, as well as the expression of ICD regulators among the clustering subtypes, were evaluated.

### Characterization of tumor immune microenvironments of different clusters

The ‘estimate’ R package was employed to assess stromal and immune cell prevalence, while ‘CIBERSORT’ was used for detailed immune cell type quantification. Box plots for visualizing TME scores were created with ‘ggplot2’, heatmaps with ‘pheatmap’, and survival outcomes were analyzed using ‘survival’ and ‘survminer’ packages.

### Establishment and validation of the prognostic system

To determine the prognostic impact of the ICD-related DEGs, each gene’s correlation with overall survival (OS) in the tumor cohorts was examined via Univariate Cox hazard analysis. Genes identified as related to survival, with a cut-off P-value of 0.05, were selected for additional study. Tumor cases were partitioned once into training (*n* = 559) and validation (*n* = 146) sets in an 8:2 ratio using stratified random sampling by OS event status (vital status/time) and AJCC pathologic stage, with set.seed(202401). We verified the allocation by comparing event rates and stage distributions between sets.LASSO-Cox modeling and λ selection. Penalized Cox models were fit with glmnet (R) using family = “cox”, alpha = 1 (LASSO), and standardize = TRUE. The penalty parameter λ was tuned by 10-fold cross-validation with folds stratified by event status (nfolds = 10, type.measure = “deviance”). We selected λ_min (the λ yielding the lowest mean cross-validated partial-likelihood deviance); λ_1se (the largest λ within one standard error of the minimum) was additionally reported to illustrate the parsimony–fit trade-off. The final coefficients were taken from the λ_min solution. .The risk score was calculated using the following formula:

$$riskScore\,=\,\sum\limits_{{i=1}}^{n} {Coef\left( {gen{e_i}} \right)} \, * \,Expression\left( {gene} \right)$$

(Coef (genei): coefficients, Expression (genei): gene expression level)

Individuals in the training cohort were categorized into low- and high-risk groups according to the median risk score, and their OS times were compared using Kaplan–Meier analysis. For validation, the verifying cohort was similarly stratified into low- and high-risk categories, and the gene model was evaluated. Clinical information, including age, gender, and tumor stage, was collected from the TCGA and GEO cohorts. These factors, along with the risk score, were analyzed using both Univariate and multivariable Cox regression models.

### The receiver operating characteristic (ROC) curve

The receiver operating characteristic (ROC) curve, which plots test sensitivity on the y-axis against 1-specificity (false positive rate) on the x-axis, serves as a powerful tool for assessing the performance of diagnostic tests. The area-under-the-curve (AUC) is the primary metric derived from the ROC curve, representing a concordance index or c-statistic. The AUC quantifies the likelihood that a randomly chosen patient who experienced an event received a higher risk score than one who did not. This metric ranges from 0.5 (indicating no discrimination, akin to a coin flip) to 1 (indicating perfect discrimination). Typically, an AUC of 0.5 suggests no discriminatory power, 0.7–0.8 is deemed acceptable, 0.8–0.9 is considered excellent, and values above 0.9 are regarded as outstanding.

### Nomogram

Additionally, we constructed a nomogram to estimate the survival probabilities at 1, 3, and 5 years, integrating the risk score as a key prognostic factor. By integrating prognostic and clinical characteristics, we utilized the R package “RMS” to construct the nomogram. Calibration curves were employed to assess the nomogram’s accuracy.

Assessment of independence and interactions. To determine whether the prognostic risk group provides information beyond clinical factors, we fit multivariable Cox models including risk group (high vs. low), AJCC pathologic stage (Stage III–IV vs. Stage I–II), age (> 65 vs. ≤ 65), and sex (male vs. female). We formally tested interactions between the risk group and each clinical factor (risk × stage, risk × age, risk × sex) and report Wald p-values as well as likelihood-ratio tests (LRTs) comparing models with versus without the interaction terms. We present adjusted hazard ratios (HRs) with 95% confidence intervals and two-sided p-values.

### Experimental validation

#### Materials

A non-targeting shRNA lentiviral construct (shNC; pSLenti-U6-shRNA(NC)-CMV-EGFP-F2A-Puro-WPRE, construct GL427NC) was used as the essential negative control in parallel with the shRNA targeting KCNN4 (shKCNN4; pSLenti-U6-shRNA(KCNN4)-CMV-EGFP-F2A-Puro-WPRE, construct Y28707). Unless otherwise specified, experimental comparisons were made between shKCNN4 and the negative-control shNC groups. Throughout all downstream assays related to Fig. 10 and associated experiments, shNC served as the reference group. PC9 cells were acquired from the Cell Resource Center, Basic Medical Sciences Institute, Peking Union Medical College, Chinese Academy of Medical Sciences. We commissioned short tandem repeat (STR) profiling and mycoplasma testing from a certified third-party vendor, Suzhou Haixing Biotechnology Co., Ltd. (Suzhou, China). Polybrene: Sigma, product number H9268.Puromycin: Amresco, product number J593.Fetal Bovine Serum (FBS): GIBCO, product number 10270-106.DMEM high glucose medium: GIBCO, product number C11965500BT.

#### Sh KCNN4

PC-9 cells were plated at a density of 1 × 10^5 cells/mL in 6-well plates, with 2 ml of cell suspension per well. After 12 to 20 h, PC-9 cells were exposed to increasing concentrations of puromycin (ranging from 0.5 µg/mL to 5 µg/mL), and cell viability was assessed over several days. Based on these experiments, 2 µg/mL was identified as the optimal concentration, as it effectively eliminated non-transfected cells within 3–5 days while maintaining the viability of transfected cells. Following another 12 to 20 h, the medium was refreshed with new culture medium. Subsequently, 2 µg/mL Puromycin was used for selection, with the Puromycin-containing medium being refreshed every 2 to 3 days until stable cell lines expressing the target gene were obtained.

#### RNA extraction and real-time PCR

Sample Preparation and RNA Extraction: The total RNA from PC-9 cell samples was isolated using Trizol reagent for resuspension. RNA Reverse Transcription and cDNA Synthesis: Genomic DNA was removed using TAKARA’s gDNA Eraser Kit, followed by cDNA synthesis using Prime Script RT enzyme. Real-time Quantitative PCR (qPCR): qPCR was performed using SYBR Green I, with specific primers for KCNN4: ATGTGGGGCAAGATCGTCTG (forward), CTTCTTGTAGCACTCGGGCA (reverse); GAPDH: GTCTTCACCACCATGGAGAA (forward), TAAGCAGTTGGTGGTGCAG (reverse). Relative gene expression levels were calculated using the 2^-ΔΔCt method.

#### Cell proliferation assay

Cell proliferation was assessed using the CCK-8 assay. The cells were seeded into 96-well plates at a concentration of 5 × 10^4 cells/mL, with each well containing 100 µL of the cell suspension. Following a 24-hour period for attachment, cell viability was evaluated on days 1, 2, and 3. At the designated time points, 10 µL of CCK-8 reagent was introduced to each well, and the plates were incubated for 1 h at 37 °C with 5% CO_2_. After incubation, optical density was measured at 450 nm with a microplate reader, and background absorbance at 650 nm was subtracted. All experiments were conducted in triplicate and replicated three times to ensure consistency.

#### Wound healing assay

Cell migration was quantified using a scratch assay. Cells were grown in 6-well plates until they reached 90% confluence. A sterile 200 µL pipette tip was then employed to create a linear scratch. After washing with PBS to remove cell debris, the wells were replenished with fresh medium. Photographs of the initial wound (0 h) and the wound at 48 h post-scratch were taken using an inverted microscope with a digital camera. In ImageJ/Fiji, wounds were segmented with the MRI Wound Healing Tool to derive the wound area and widths, with the healing rate calculated using the formula:

$$Healing~Rate\left( \% \right)=\,\frac{{Initial~Wound~Area - Wound~Area~at~48~h}}{{Initial~Wound~Area}}\, * \,100$$

#### Cell invasion assays

For the Transwell invasion assay, 24-well Transwell inserts with 8-µm pores (polycarbonate membrane) were pre-coated with growth factor–reduced Matrigel kept on ice. Matrigel stock ( protein concentration : 8 mg/mL) was diluted 1:8 (v/v) in pre-chilled serum-free medium to yield a 1 mg/mL working solution. A volume of 50 µL of the diluted Matrigel was added to the upper surface of each insert and allowed to polymerize at 37 ℃ for 30 min. After gelation, 200 µL of a cell suspension (3 × 10^5 cells/mL in serum-free medium) was gently added to the upper chamber. The lower chamber was filled with 600 µL of complete medium containing 20% fetal bovine serum (FBS) as a chemoattractant, while the upper chamber contained 0% FBS to maintain the serum gradient. Cells were incubated for 48 h at 37 °C in a humidified atmosphere with 5% CO₂. Non-invading cells remaining on the upper membrane surface were removed with a cotton swab. Membranes were fixed in 4% paraformaldehyde for 15 min at room temperature, stained with 0.1% (w/v) crystal violet for 20 min, and rinsed thoroughly with PBS. Invaded cells adherent to the lower membrane surface were imaged and counted under a light microscope at 200×magnification in at least five randomly selected fields per insert. The invading cells are counted under a microscope in at least five random fields per membrane.

#### Molecular Docking and molecular dynamics simulations

This study employed the Schrödinger software suite for virtual screening, targeting 3,330 FDA-approved small molecule compounds from the ZINC database.Library-level inclusion criteria were: molecular weight 180–750 Da; cLogP (XlogP3) between − 1 and 7; H-bond donors ≤ 5; H-bond acceptors ≤ 10; rotatable bonds ≤ 12; topological polar surface area ≤ 140 Å²; formal charge between − 2 and + 2 at pH 7.4. Initially, the LigPrep module was utilized to perform energy minimization and protonation state prediction under the *OPLS4* force field. The OPLS4 (Optimized Potentials for Liquid Simulations) force field is a widely used computational model for simulating molecular interactions. It is designed to predict the behavior of molecules by accounting for forces between atoms, such as bond stretching, angle bending, and van der Waals forces. In this study, we employed the OPLS4 force field during energy minimization and protonation state prediction to ensure accurate modeling of the small molecules and protein, which is essential for reliable virtual screening results. Subsequently, the crystal structure of the KCNN4 protein (PDB ID: 6cnm) was downloaded from the PDB database. The 6CNM crystal structure was imported into Maestro and prepared with Protein Preparation Wizard at pH 7.4: bond orders were assigned; hydrogens were added; zero-occupancy/duplicate altLocs were pruned; the highest-occupancy alternate conformer per residue was retained; and disulfide bonds were detected and assigned automatically.Following this, the Receptor Grid Generation module, along with the sitemap-predicted binding sites, was employed to generate a docking grid box with dimensions of 25 × 25 × 25 Å³. Finally, the SP algorithm in the Glide module was applied to conduct semi-flexible docking calculations, allowing the identification of potential KCNN4-targeting compounds based on docking scores.

All-atom molecular dynamics simulations of small molecule-protein complexes were conducted using AMBER 20 software [17]. Charges for small molecules were derived via Hartree-Fock SCF/6-31G* calculations using Gaussian 09 and the antechamber module. The GAFF2 and ff14SB force fields were applied to small molecules and proteins, respectively [18, 19]. Systems were solvated in a 10 Å TIP3P water box with Na+/Cl- ions for neutralization6. Energy minimization consisted of 2500 steps each of steepest descent and conjugate gradient methods. Systems were heated from 0 K to 298.15 K over 200 ps at constant volume, followed by 500 ps NVT and NPT equilibration. 100 ns NPT production simulations were performed under periodic boundary conditions. Non-bonded interactions were constrained to a 10 Å cutoff, with long-range electrostatics calculated via the Particle Mesh Ewald (PME) approach. The PME method is a mathematical technique used in molecular dynamics simulations to efficiently calculate long-range electrostatic interactions, which are critical for maintaining accurate system dynamics over time. These long-range forces, particularly in systems like proteins and small molecules, can significantly influence molecular behavior and interactions. In our simulations, PME was employed to ensure precise calculation of electrostatic forces, contributing to the accuracy of the molecular dynamics simulations over the 100 ns timescale. Hydrogen bond lengths were maintained using the SHAKE algorithm. The SHAKE algorithm is used to constrain the lengths of hydrogen bonds during molecular dynamics simulations. Hydrogen atoms, due to their small mass, can introduce instabilities in the simulation if allowed to vibrate freely. By applying the SHAKE algorithm, we fix the hydrogen bond lengths, allowing for a larger integration time step (2 fs) without compromising the stability of the simulation. This technique enables us to efficiently simulate longer time periods while ensuring accurate representation of the molecular interactions. A Langevin collision frequency (γ) of 2 ps⁻¹ was chosen as a standard compromise that provides efficient temperature control in explicit solvent while minimally perturbing configurational dynamics for solvated proteins; this value lies within the 1–5 ps⁻¹ range commonly recommended for biomolecular simulations in AMBER and is widely used in practice for 100-ns–scale trajectories .Trajectories for analysis were collected every 10 ps.

### Statistical analysis

Continuous variables are summarized as mean ± SD (if approximately normal) or median (IQR) otherwise; categorical variables as counts (percentages).The Kaplan–Meier survival analysis was executed with the survival and survminer packages. Student t tests were used to compare differences between two groups, while ANOVA tests were employed for comparisons among three or more groups. For non-normally distributed data, the Wilcoxon rank-sum test (for two groups) or the Kruskal–Wallis test (for multiple groups) was applied. Associations between groups were evaluated using Pearson’s chi-square (χ²) test. For 2 × 2 tables a Yates continuity correction was applied. When any expected cell count was < 5, we confirmed inferences using χ² with Monte-Carlo simulated exact P-values (10,000 replicates) or Fisher’s exact test, as appropriate. A threshold p-value of less than 0.05 was used to determine statistical significance. To localize which cells drove any significant global association, we computed cellwise standardized Pearson residuals (SR):

$$S{R_{ij}}\,=\,\frac{{{O_{ij}} - {E_{_{{ij}}}}}}{{\sqrt {{E_{ij}}} }}$$      

 where *O*_*ij*_is the observed count and E_*ij*_$\:=\frac{\mathbf{R}\mathbf{o}\mathbf{w}\boldsymbol{i}\mathbf{*}\mathbf{C}\mathbf{o}\mathbf{l}\boldsymbol{j}}{\mathbf{N}}$ is the expected count under independence. By convention,∣SR∣≥2 indicates a notable cell-level deviation (SR > 0 enrichment; SR < 0 depletion)

## Results

**Fig. 1:**
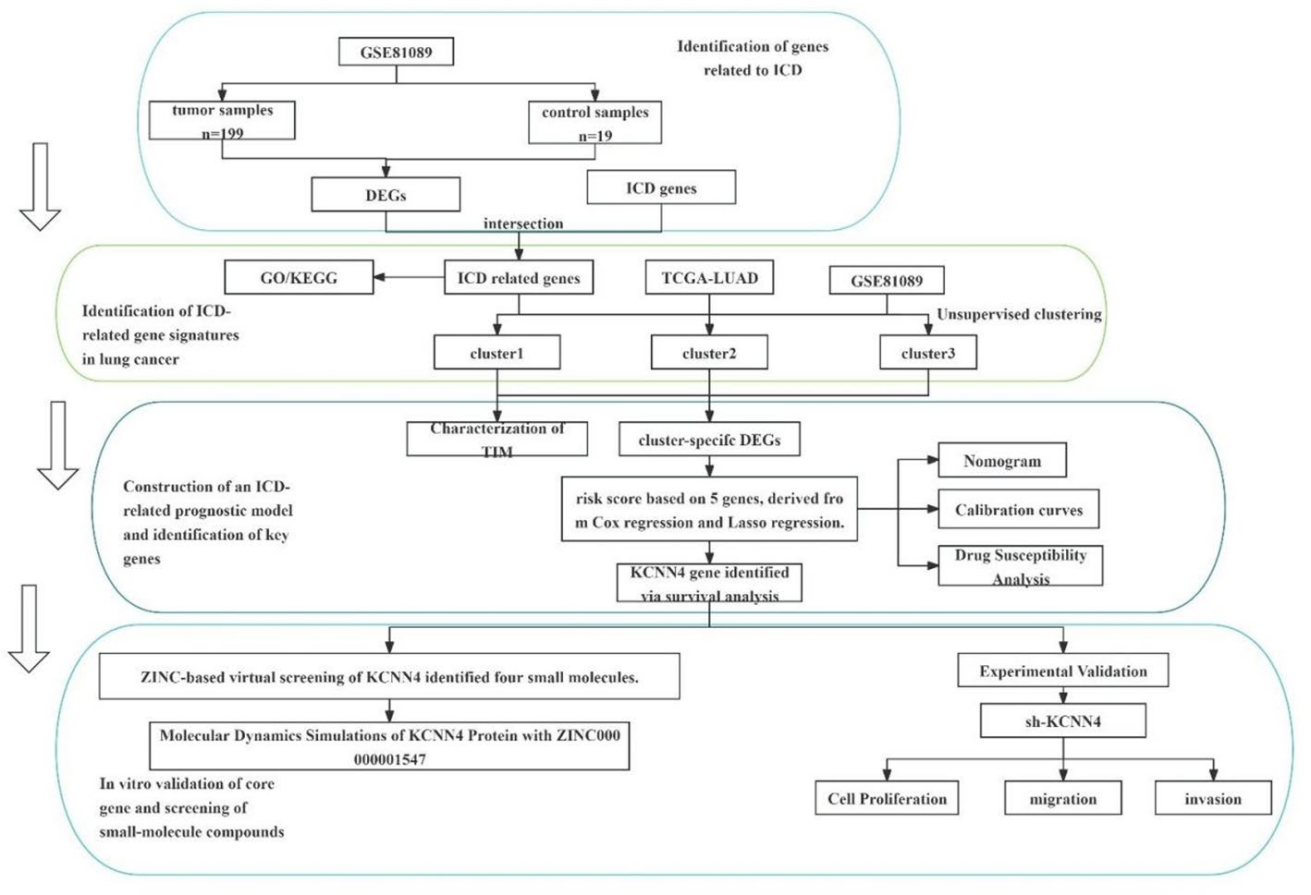
The workflow of the study .*DEGs* Differentially Expressed Genes, *GO*༚Gene Ontology, *KEGG*༚Kyoto Encyclopedia of Genes and Genomes, *ICD*༚Immunogenic Cell Death, *TCGA-LUAD*༚The Cancer Genome Atlas - Lung Adenocarcinoma, *TIM*༚Tumor Immune Microenvironment, *sh-KCNN4*༚Short Hairpin RNA targeting KCNN4

The overall research workflow of this study is illustrated in Fig 1.

### Immunogenic cell death-related DEGs identification

**Fig. 2:**
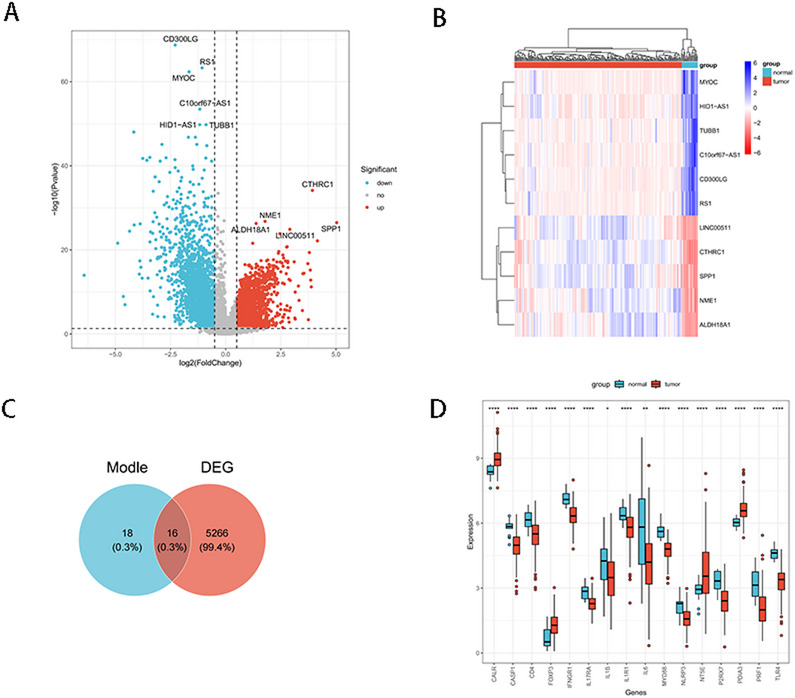
Differential expression analysis of ICD-related DEGs. **A** Volcano plot. The x-axis shows log₂(fold change) for tumor vs. control; positive values indicate higher expression in tumor, negative values indicate lower expression in tumor. Red dots = upregulated DEGs (tumor-high), blue dots = down regulated DEGs (tumor-low), gray = not significant at FDR 0.05. **B** Heatmap of top DEGs. Rows represent genes and columns represent individual samples (tumor or control). Red = higher, blue = lower. **C** Venn diagram. Overlap between the pre-compiled ICD gene set and the DEG list derived from panel A (FDR < 0.05, |log₂FC| ≥ 1.0). **D** Box plot of ICD-related DEGs in the GEO dataset between the tumor and the control groups. Asterisks represented p-value (****p < 0.0001, ***p < 0.001, **p < 0.01, *p < 0.05.)

Comparison of tumor samples with healthy controls in the GSE81089 cohort identified 5282 differentially expressed genes (DEGs) that were significantly distinct between the two groups. Of these, 2733 genes were upregulated, while 2549 were downregulated (Table S1). The DEGs were depicted in a volcano plot (Fig. 2A). Furthermore, the DEGs with the smallest P values, consisting of 5 upregulated and 6 downregulated genes, were presented in a heatmap (Fig. 2B). From the overlap of DEGs and ICD-related genes (Table S2), we identified 16 ICD-related DEGs, including NT5E, CALR, PDIA3, IL6, CASP1, IL1R1, IL1B, NLRP3, P2RX7, MYD88, TLR4, CD4, FOXP3, IFNGR1, IL17RA, PRF1(Fig. 2C).In order to explore the expression differences of 16 ICD-related DEGs in the lung cancer dataset, a boxplot was drawn (Fig. 2D). All the ICD-related DEGs were significantly different between the tumor group and the control group (P value < 0.05). Compared with the normal tissue group, the expression of ICD-associated genes, including HMGB1, ATP, CALR, IL-1β, and CXCL10, was significantly upregulated in the lung cancer group. Elevated HMGB1 and ATP levels suggest enhanced release of immunogenic signals capable of activating dendritic cells and macrophages, potentially promoting anti-tumor immunity. Overexpression of CALR indicates increased exposure of “eat-me” signals on the tumor cell surface, potentially facilitating immune cell recruitment. Additionally, the upregulation of IL-1β and CXCL10 may reflect an inflammatory microenvironment that enhances T cell activation and recruitment. These findings suggest that the increased expression of ICD-related genes in lung cancer may contribute to tumor immune responses and provide potential targets for immunotherapeutic strategies.

### Enrichment analyses (GO/KEGG)

**Fig. 3:**
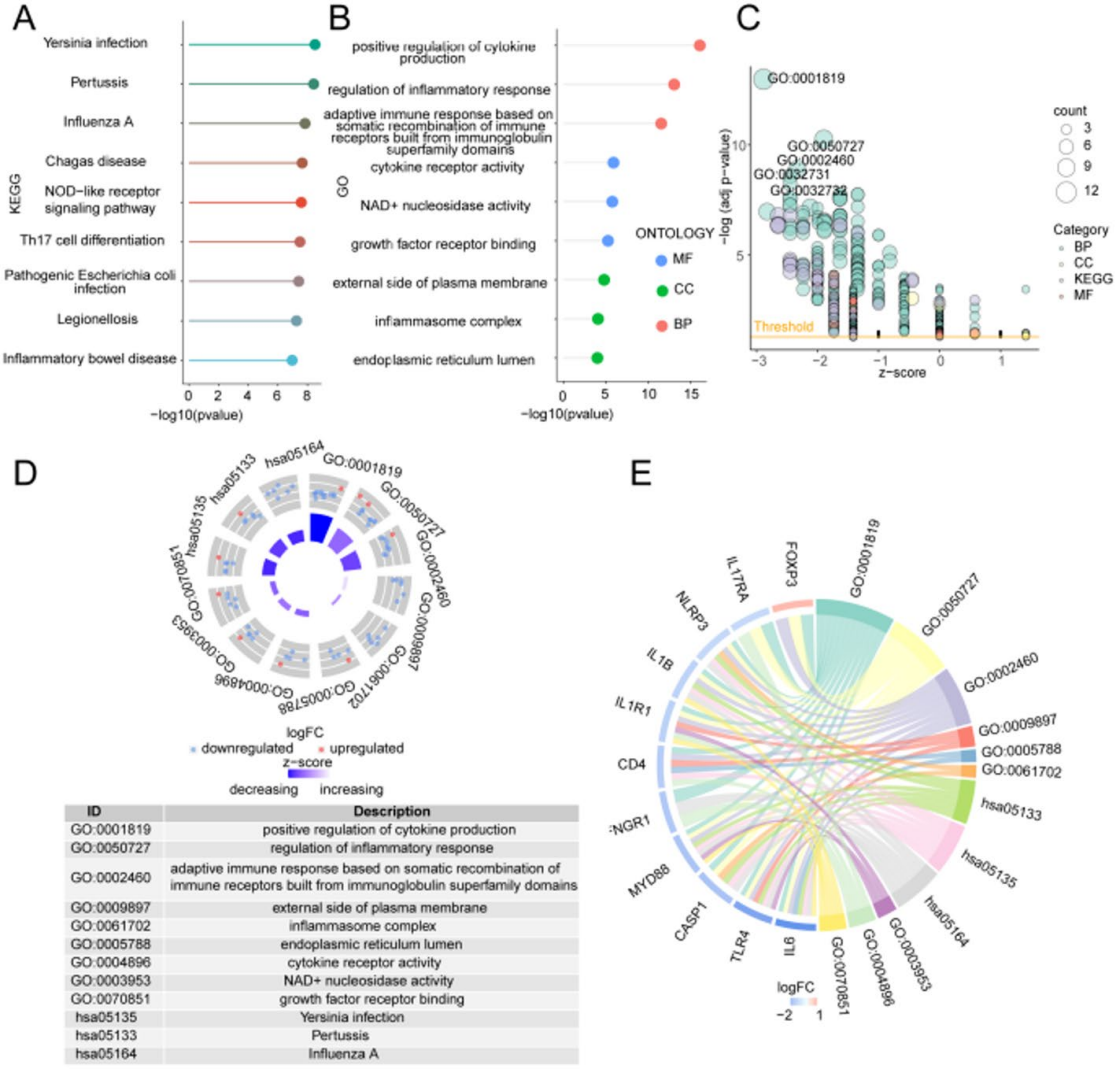
GO and KEGG enrichment analysis. **A** KEGG enrichment analysis for the ICD-related DEGs showing the significant terms. **B** GO enrichment analysis for the ICD-related DEGs showing the significant terms. **C** GO enrichment analysis bubble map, horizontal coordinate is z-score score, bubble size indicates the number of genes enriched to the GO term. **D** Circle plot visualizing the pathways enriched by gene ontology (GO) and KEGG analysis, the outer circle is scatter plot, each dot represents a gene, red represents gene up-regulation; The inner circle is the bar chart, where the height represents significance, and the color represents z-score. The bluer it is, the more down-regulated genes are enriched. **E** GO and KEGG enrichment analyses of ICD-related DEGs. with genes on the left and pathways on the right. *GO* Gene Ontology, *BP* biological process, *CC* cellular component, *MF* molecular function, *KEGG* The Kyoto Encyclopedia of Genes and Genomes. The screening criteria for GO and KEGG enrichment items were *P* < 0.05

Sixteen ICD-related DEGs (NT5E, CALR, PDIA3, IL6, CASP1, IL1R1, IL1B, NLRP3, P2RX7, MYD88, TLR4, CD4, FOXP3, IFNGR1, IL17RA, PRF1) were selected as key genes in this study to investigate the relationship between biological pathways and non-small cell lung cancer. GO and KEGG enrichment analysis was performed for key genes (Table S3-4). The key genes were primarily enriched in biological processes (BP) such as the positive regulation of cytokine production, the regulation of inflammatory responses, and adaptive immune responses mediated by somatic recombination of immune receptors derived from immunoglobulin superfamily domains, external side of plasma membrane, inflammasome complex, endoplasmic reticulum lumen and other cellular components (CC), cytokine receptor activity, NAD+ nucleosidase activity, growth factor receptor binding and other molecular functions (MF), Yersinia infection, Pertussis, Influenza A, Chagas disease, NOD−like receptor signaling pathway, Th17 cell differentiation, Pathogenic Escherichia coli infection, Legionellosis, Inflammatory bowel disease and other pathways (Fig. 3A-E).

### Novel subtypes of lung cancer identified by unsupervised clustering

**Fig. 4:**
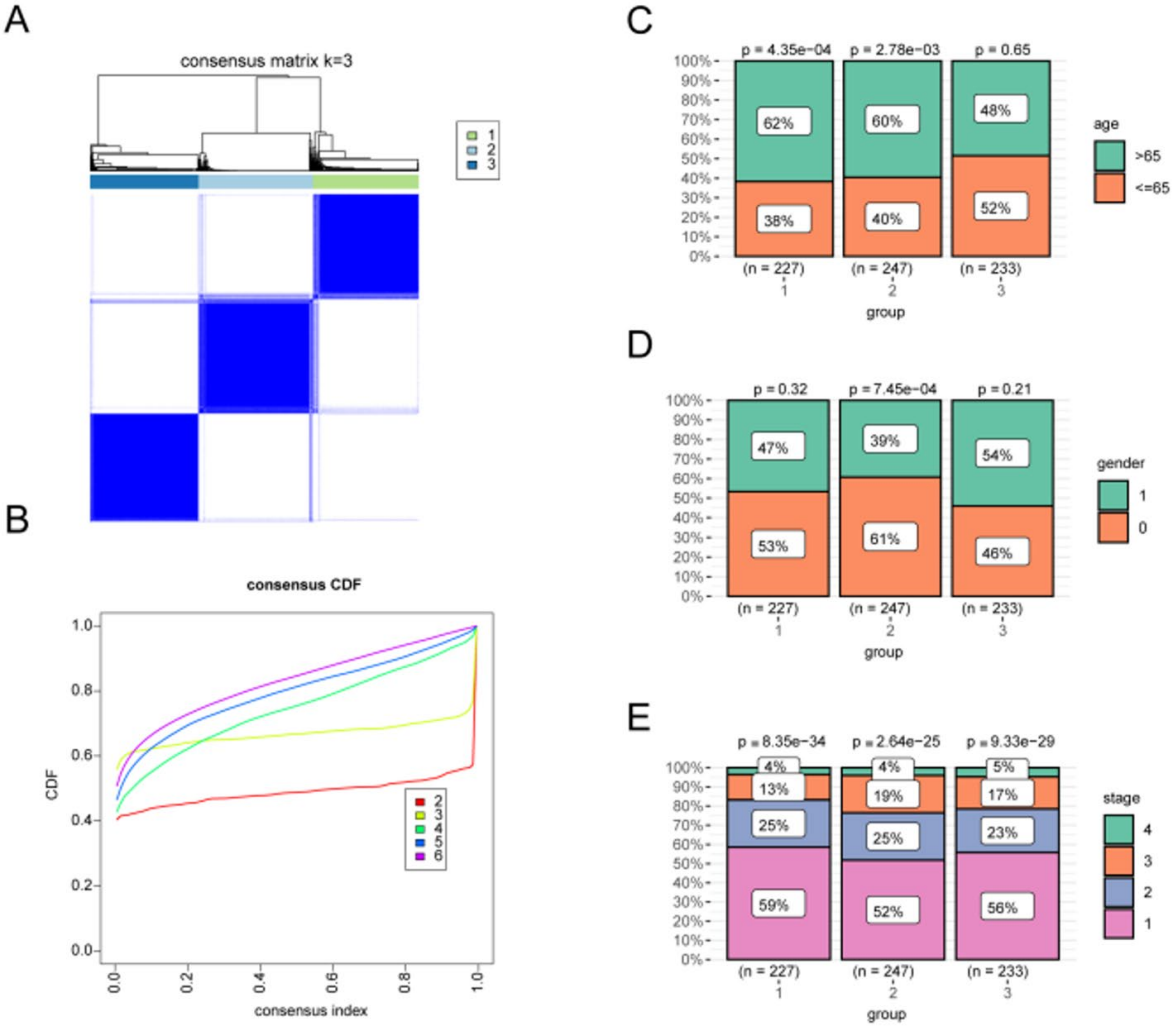
Unsupervised consensus clustering of ICD-related gene expression in LUAD. **A** Consensus matrix for k = 3 under 1 − Pearson distance, average-linkage hierarchical clustering with reps = 1000, pItem = 0.8, pFeature = 1.0. **B** CDF and ΔCDF across k = 2–6; k = 3 was selected based on ΔCDF plateau and minimal PAC, with cluster-level consensus ≥ 0.80 and median item-level consensus ≥ 0.60 and no clusters < 5% of samples. **C**–**E** Associations between consensus subtypes and clinical features

The gene expression profiles of ICD-associated genes were used to investigate the lung cancer subtypes from the tumor cohorts. Based on the consensus matrix distribution (Fig. 4A) and the cumulative distribution function (CDF) (Fig. 4B), the optimal number of clusters was determined to be k = 3.Using an unsupervised clustering analysis, three distinct modification patterns were identified, designated as Cluster 1, Cluster 2, and Cluster 3. .Age distribution varied across subtypes (χ²= 10.39, *P* = 0.0055). Within-subtype percentages showed a larger proportion of patients < 65 years in Cluster 3 (51.9%) compared with Cluster 1 (38.3%) and Cluster 2 (40.1%). Standardized Pearson residuals (SR) suggested a mild enrichment of < 65 years in Cluster 3 (SR = + 1.97) with a reciprocal depletion of ≥ 65 years (SR = − 1.73); residuals in other cells were smaller in magnitude (|SR|<2)(Fig. 4C) Sex distribution also differed significantly (χ²= 11.199, *P* = 0.0037, small effect). Females were proportionally higher in Cluster 2 (61.1%) and lower in Cluster 3 (45.9%), with the opposite pattern for males (Cluster 3: 54.1% vs. Cluster 2: 38.9%). (Fig. 4D) Stage distribution did not differ across subtypes (χ²= 4.368, *P* = 0.627). All cellwise residuals were within |SR|<2, indicating no cell-level departures from independence.(Fig. 4E).

### Characterization of tumor immune microenvironments of three clusters

**Fig. 5:**
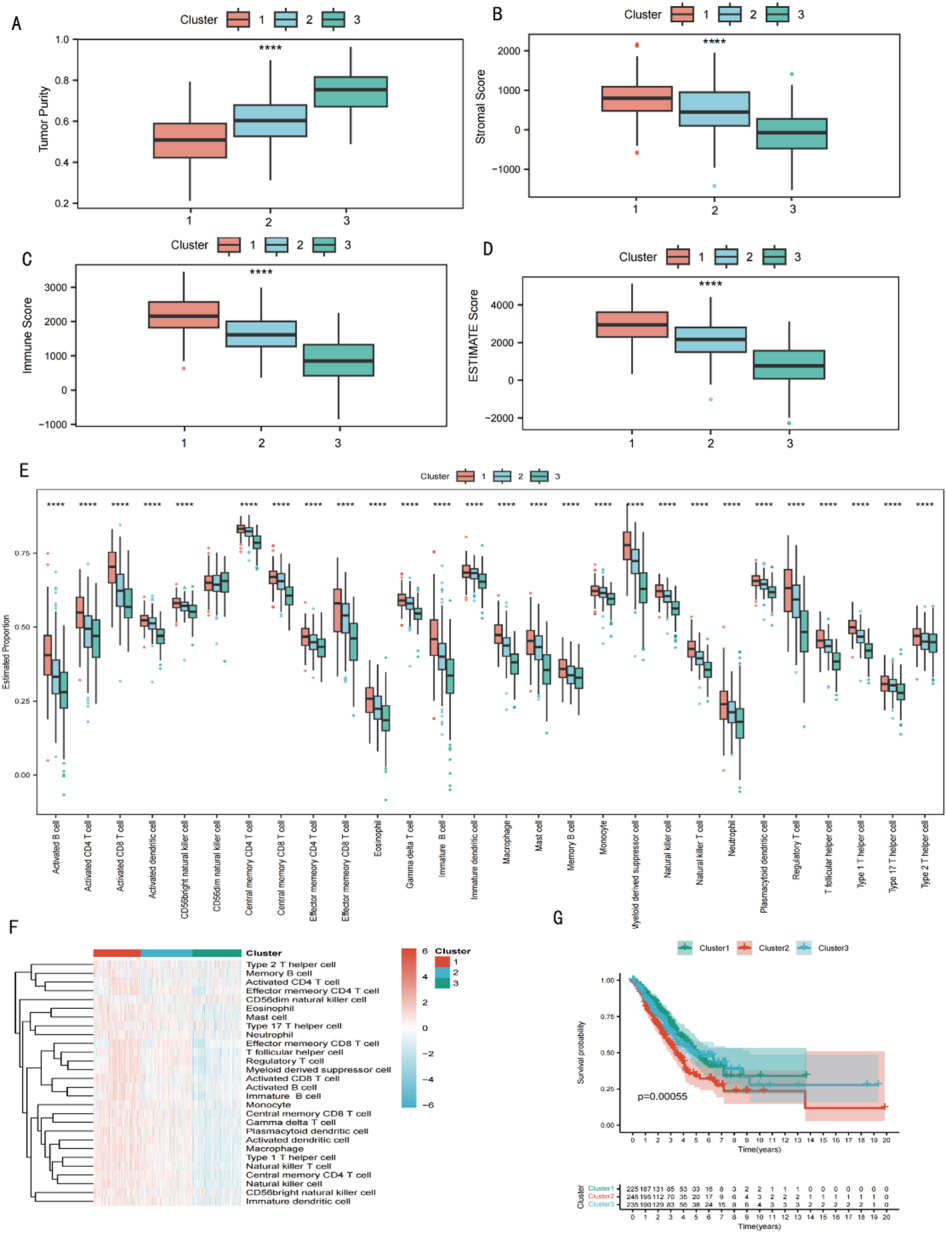
Characterization of Tumor Immune Microenvironments in Three Distinct Clusters. **A** Box plot illustrating tumor purity across Clusters 1, 2, and 3. **B** Box plot of stromal scores for each cluster. **C** Box plot of immune scores for each cluster. **D** ESTIMATE scores for the clusters indicating the level of stromal and immune cell infiltration in tumor tissues. **E** Swarm plot showing the distribution of various immune cell types within each cluster. **F** Heatmap representing the expression levels of immune cell types across the three clusters. **G** Kaplan-Meier survival curves for the clusters, comparing overall patient survival. .****p < 0.0001, ***p < 0.001, **p < 0.01, *p < 0.05

We compared tumor purity, immune scores, and stromal scores among three tumor clusters. The analysis yielded significant results, demonstrating that Cluster 3 had a notably higher median tumor purity compared to Clusters 2 and Cluster 1 (*p* < 0.0001) Specifically, tumor purity differed markedly (Cluster 1: 0.50 ± 0.11; Cluster 2: 0.60 ± 0.11; Cluster 3: 0.75 ± 0.10). Pairwise contrasts (Holm-adjusted) showed Cluster 3 higher than both Cluster 1 and Cluster 2 (C3 vs. C1, *p* < 0.0001; C3 vs. C2, *p* < 0.0001), with Cluster 2 also higher than Cluster 1 (C2 vs. C1, *p* < 0.0001). The median tumor purity in Cluster 3 was approximately 26% higher than in Cluster 1 and 18% higher than in Cluster 2 (*p* < 0.0001) (Fig. 5A), suggesting a higher proportion of malignant cells. However, further analysis of other markers, such as proliferation indices, invasion-related gene expression, and clinical outcomes (e.g., metastasis rates), is necessary to more accurately assess the malignancy and invasiveness of tumors in this cluster. Regarding immune scores, Cluster 1 exhibited a significant increase relative to Clusters 2 and 3 (*p* < 0.0001) (Fig. 5C).Cluster 1 was clearly highest (Cluster 1: 2169.79 ± 511.54; Cluster 2: 1637.34 ± 507.27; Cluster 3: 826.89 ± 610.84).Quantification reveals that the immune score in Cluster 1 was approximately 35% higher than in Cluster 2 and 40% higher than in Cluster 3 (*p* < 0.0001), suggesting a highly active infiltration of immune cells in its tumor microenvironment, which may potentially correlate with a favorable response to immunotherapy. Conversely, stromal scores were lowest in Cluster 3 (Cluster 1: 777.54 ± 508.07; Cluster 2: 505.64 ± 579.01; Cluster 3: − 100.71 ± 567.71). Cluster 3 was lower than both Cluster 1 and Cluster 2 (C3 vs. C1, *p* < 0.0001; C3 vs. C2, *p* < 0.0001)(Fig. 5B), indicating a marked depletion of connective-tissue elements and weaker extracellular-matrix interactions in Cluster 3.Specifically, the stromal score in Cluster 3 was 42% lower than in Cluster 1 and 36% lower than in Cluster 2 (*p* < 0.0001), leading to notably weakened extracellular matrix interactions, which are directly related to the stability of tumor structure and its response to chemotherapy drugs.

Upon subdivision and proportional estimation of immune cells within tumor samples, our population dynamics analysis identified three unique immune microenvironments (Clusters 1, 2, and 3), each characterized by a distinct immune cell composition. Cluster 1 was significantly enriched with effector immune cells. The proportions of activated CD4^+^ T cells( Cluster 1:0.54 ± 0.07,Cluster 2: 0.48 ± 0.08, Cluster 3༚0.46 ± 0.08, C1 vs. C2, *p* < 0.0001; C1 vs. C3, *p* < 0.0001), activated CD8^+^ T cells(Cluster 1 ༚0.69 ± 0.07, Cluster 2༚0.62 ± 0.07, Cluster 3༚0.57 ± 0.07, C1 vs. C2, *p* < 0.0001; C1 vs. C3, *p* < 0.0001༉, central memory CD4^+^ T cells༈Cluster1༚0.83 ± 0.02, Cluster 2༚0.82 ± 0.02, Cluster 3༚0.78 ± 0.03, C1 vs. C2, *p* < 0.0001; C1 vs. C3, *p* < 0.0001༉, effector memory CD4^+^ T cells༈Cluster 1༚0.46 ± 0.04, Cluster 2 0.45 ± 0.04, Cluster 3 0.43 ± 0.04, C1 vs. C2, *p* < 0.0001; C1 vs. C3, *p* < 0.0001༉, effector memory CD8^+^ T cells༈Cluster 1༚0.55 ± 0.09, Cluster 2༚0.52 ± 0.08, Cluster 3༚0.44 ± 0.08༉ and CD56 bright natural killer cells ༈Cluster 1 0.62 ± 0.03, Cluster 2 0.60 ± 0.03, Cluster 3 0.56 ± 0.04, C1 vs. C3, *p* < 0.0001; C2 vs. C3, *p* < 0.0001༉were notably elevated (*p* < 0.001) (Fig. 5E). This cellular configuration suggests an inflamed immune microenvironment with a heightened cell-mediated immune response, potentially correlating with robust anti-tumor activity. Conversely, Cluster 2 exhibited an immune-desert tendency, with fewer effector memory T cells—effector memory CD4^+^(Cluster 2 0.45 ± 0.04 vs. Cluster 1 0.46 ± 0.04, *p* < 0.0001) and effector memory CD8^+^(Cluster 2 0.52 ± 0.08vs Cluster 1 0.55 ± 0.09, *p* < 0.0001)—and a notable decline in γδ T cells (Cluster 2 0.57 ± 0.03 vs. Cluster 1 0.59 ± 0.03, *p* < 0.0001; Cluster 3 0.54 ± 0.03vs Cluster 2, *p* < 0.0001). This trend indicates an immune desert microenvironment likely characterized by low responsiveness to immunotherapeutic interventions. Finally, Cluster 3 showed broad reductions in effector populations consistent with an immunosuppressive milieu. In particular, activated CD4^+^T cells (Cluster 3 0.46 ± 0.08vs Cluster 1 0.54 ± 0.07, *p* < 0.0001), activated CD8^+^T cells (Cluster 3 0.57 ± 0.07 vs. Cluster 1 0.69 ± 0.07, *p* < 0.0001), and CD56^bright NK cells (Cluster 3 0.56 ± 0.03 vs. Cluster 1 0.62 ± 0.03, *p* < 0.0001) were all significantly lower. (Fig. 5E), possibly denoting an immunosuppressive microenvironment where the tumor’s capability to evade immune surveillance is enhanced. Lastly, the survival outcomes associated with these immune microenvironments were clearly delineated by the K-M survival curves in Fig. 5G. Overall survival differed significantly among the three clusters (log-rank χ²=776.36, df = 2, *p* < 0.0001).Median survival (years) was 4.93 in Cluster 1, 3.46 in Cluster 2, and 4.73 in Cluster 3.Pairwise log-rank tests (Holm-adjusted) confirmed significant differences for Cluster 1 vs. 2 (*p* < 0.0001), Cluster 1 vs. 3 (*p* < 0.0001), and Cluster 2 vs. 3 (*p* < 0.0001).Here, a significant difference in OS among the clusters is shown, with patients in Cluster 1 exhibiting a survival benefit. Conversely, Cluster 2 is associated with the poorest prognosis.

### Construction and validation of prognostic model

**Fig. 6:**
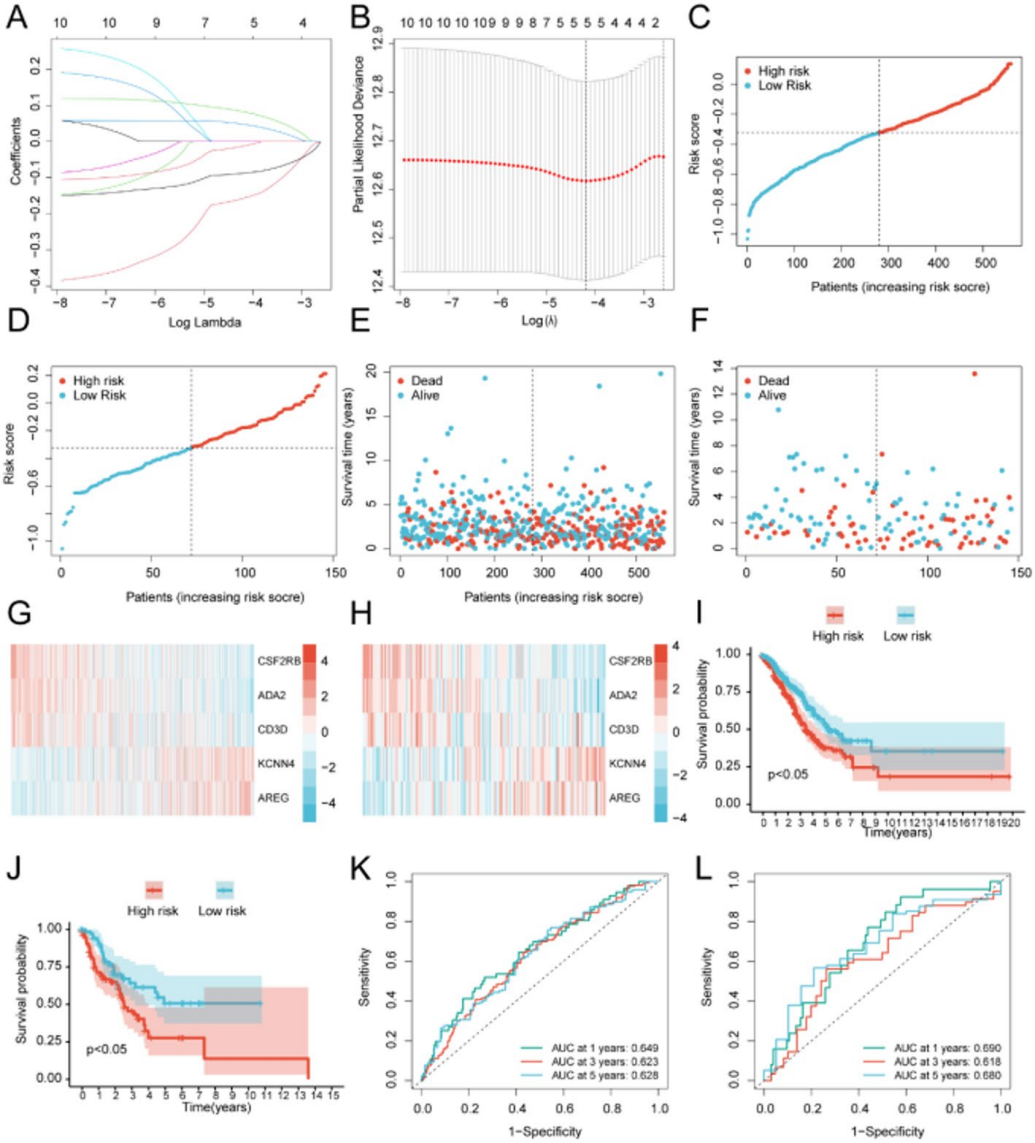
Creation and validation of a prognostic risk model. **A**, **B** Lasso regression analysis of DEGs linked to prognosis. **C** Survival analysis in the training cohort. **D** Survival analysis in the validation cohort. **E** Distribution of risk scores in the training cohort. **F** Distribution of risk scores in the validation cohort. **G** Heatmap of key genes in the training cohort. **H** Heatmap of key genes in the validation cohort. **I** Survival analysis comparing low- and high-risk groups in the training cohort. **J** Survival analysis comparing low- and high-risk groups in the validation cohort. **K** The ROC curve of the training set. **L** The ROC curve of the validation set

To evaluate the prognostic significance of 49 DEGs across the tumor clusters (Table S5), we selected genes based on their differential expression and relevance to immune modulation and tumor progression. These genes were not limited to ICD-related DEGs but were identified as significant survival-related markers through univariate Cox regression analyses.This analysis identified 10 genes (IL10RA, CSF2RB, TAGAP, CYTIP, PLEK, PTPRC, ADA2, CD3D, KCNN4, AREG) associated with overall survival (OS) in patients. For developing a more clinically applicable scoring model, we applied Lasso regression analysis to these survival-related DEGs (Fig. 6A and B), which narrowed down the list to 5 key genes (CSF2RB, CD3D, ADA2, KCNN4, AREG). Based on multivariate Cox regression analysis results, we then constructed a risk model using the following formula: Risk Score= (-0.157)*CSF2RB+༈-0.012༉*CD3D+༈-0.088༉*ADA2+༈0.083༉*KCNN4+༈0.048༉*AREG.

According to the median of risk score in training cohort, patients with lung cancer from training and validating cohorts were divided into low- and high-risk groups. Risk score curve (Fig. 6C-D), survival state scatter plot (Fig. 6E-F) and prognostic gene expression heatmap (Fig. 6G-H) were drawn in the training set and test set, respectively. It can be seen that the number of deaths in the high-risk group was greater than that in the low-risk group. The expression levels of KCNN4 and AREG increased with the increase of risk score, while the expression levels of CSF2RB, CD3D and ADA2 decreased with the increase of risk score. Kaplan-Meier survival curve analysis (Fig. 6I-J) and time-dependent ROC analysis (Fig. 6K-L) were performed in the training set and test set, respectively. Whether in the training set or the test set, the survival of the low-risk group was better than that of the high-risk group (*P* < 0.05). ROC results showed that this model could evaluate the performance of diagnostic tests (AUC > 0.6).

### Validation of prognostic model

**Fig. 7:**
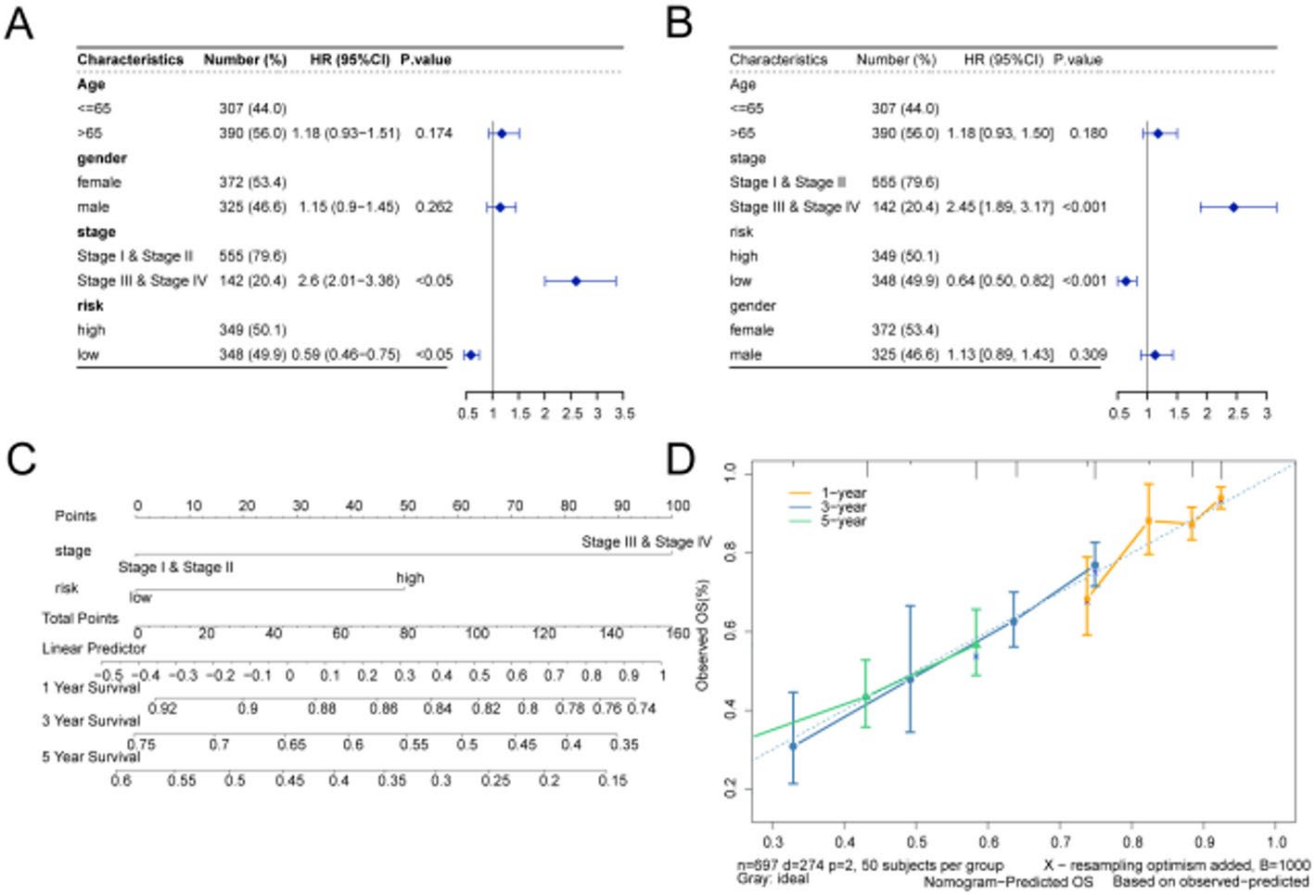
Validating of prognostic characteristics. **A** Univariate Cox analysis of forest maps combining clinical information with risk scores. **B** Multivariate Cox analysis of forest maps combining clinical information with risk scores. **C** The nomogram constructed by stage and risk predicted the survival rate of individuals at 1, 3 and 5 years. **D** Calibration curves for the validation of the nomogram

The results of the univariate Cox regression analysis indicated that the tumor staging and risk score are significantly associated with OS (*P* < 0.05, Fig. 7A). Multivariate Cox regression analysis further confirmed that tumor staging and risk score are independently associated with prognosis (*P* < 0.05, Fig. 7B). Nomograms are widely utilized in oncology to intuitively assess patient prognosis. To visually represent a statistical prognostic model estimating the probability of cancer-related death in LUAD patients, we developed a nomogram. By integrating clinico-pathological factors, including stage and ICD-related gene prognostic characteristics, the nomogram was constructed to predict 1-, 3-, and 5-year survival probabilities (Fig. 7C).

In the multivariable Cox model including stage, age, and sex, the high-risk group was significantly associated with worse overall survival compared with the low-risk group (HR = 1.60, 95% CI 1.24–2.03, *p* = 0.0002), while Stage III–IV (vs. I–II) was also significant (HR = 2.40, 95% CI 1.85–3.11, *p* < 0.0001). Age > 65 (vs. ≤ 65) and sex (male vs. female) were not statistically significant (HR = 1.20, 95% CI 0.94–1.53, *p* = 0.14; HR = 1.12, 95% CI 0.88–1.42, *p* = 0.36, respectively).Interaction tests. There was no evidence that the effect of the risk group varied by stage (risk×stage: Wald *p* = 0.47; LRT *p* = 0.47), age (risk×age: Wald *p* = 0.94; LRT *p* = 0.94), or sex (risk×sex: Wald *p* = 0.07; LRT *p* = 0.06). Collectively, these analyses demonstrate that the risk group is an independent prognostic factor after adjustment for tumor stage, age, and sex, and no significant interactions were detected with these clinical variables.

The model achieved a Harrell’s C-index of 0.60 (95% CI 0.56–0.64) in the training cohort and 0.58(95%CI 0.49–0.65) in the test cohort, indicating moderate discrimination. Calibration assessed by Hosmer-Lemeshow tests on time-specific risks was acceptable at 1 year (training *p* = 0.49, test *p* = 0.09) and 3 years (training *p* = 0.01, test *p* = 0.43), whereas 5-year calibration showed significant misfit (training *p* < 0.0001; test *p* = 0.001), suggesting the need for temporal recalibration for long-term predictions.The calibration plot validated the accuracy of the nomogram (Fig. 7D).

### Drug susceptibility analysis

**Fig. 8:**
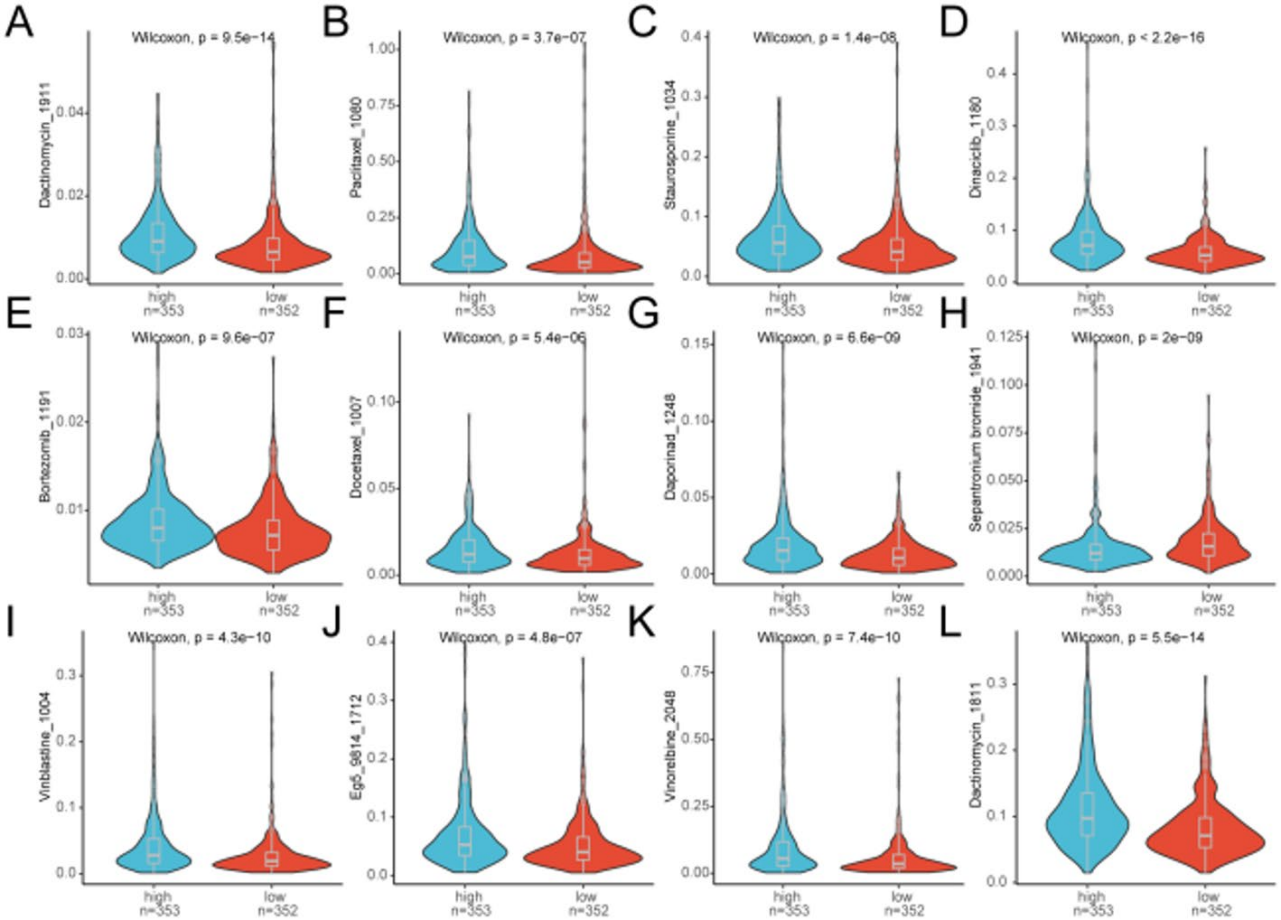
Violin plot visualizes the association between the drug susceptibility and risk scores. **A** Difference of Dactinomycin_1911 between low- and high-risk groups. **B** Difference of Paclitaxel_1080 between low- and high-risk groups. **C** Difference of Staurosporine_1034 between low- and high-risk groups. **D** Difference of Dinaciclib_1180 between low- and high-risk groups. **E** Difference of Bortezomib_1191 between low- and high-risk groups. **F** Difference of Docetaxel_1007 between low- and high-risk groups. **G** Difference of Daporinad_1248 between low- and high-risk groups. **H** Difference of epantronium bromide_1941 between low- and high-risk groups. **I** Difference of Vinblastine_1004 between low- and high-risk groups. **J** Difference of Eg5_9814_1712 between low- and high-risk groups. **K** Difference of Vinorelbine_2048 between low- and high-risk groups. **L** Difference of Dactinomycin_1811 between low- and high-risk groups

We evaluated the ability of the risk score to predict the chemotherapeutic sensitivity in lung cancer patients accurately. Dactinomycin_1911, Paclitaxel_1080, Staurosporine_1034, Dinaciclib_1180, Bortezomib_1191,Docetaxel_1007, Daporinad_1248, epantronium bromide_1941,Vinblastine_1004, Eg5_9814_1712, Vinorelbine_2048, Dactinomycin_1811 were investigated in the clinical efficiency of LUAD treatment (Fig. 8). Patients with low risk score might respond more sensitively to the Dactinomycin_1911(Fig. 8A)、Paclitaxel_1080༈Fig. 8B༉、Staurosporine_1034༈FigFig8C༉、Dinaciclib_1180༈Fig. 8D༉、Bortezomib_1191༈Fig. 8E༉、Docetaxel_1007༈Fig. 8F༉、Daporinad_1248༈Fig. 8G༉、Vinblastine_1004༈Fig. 8I༉、Eg5_9814༈Fig. 8J༉、Vinorelbine_2048༈Fig. 8K༉、Dactolisib_1811༈Fig. 8L༉, indicating that chemotherapy could be particularly effective for patients with a low risk score. Conversely, those with a high risk score might exhibit a heightened sensitivity to Sepantronium bromide _1941 (Fig. 8H).

**Fig. 9:**
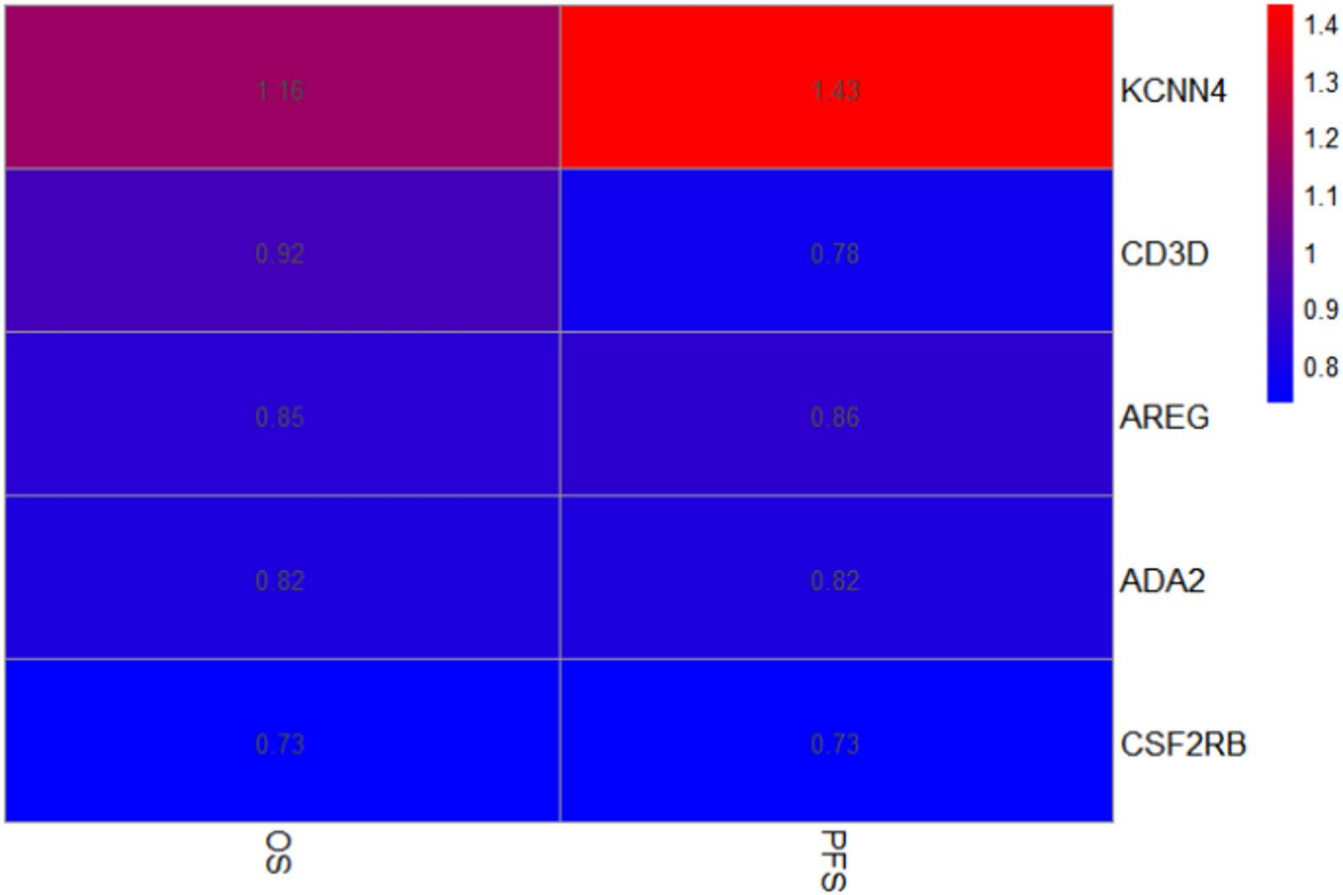
Heatmap representation of hazard ratios associated with KCNN4, CD3D, AREG, ADA2, and CSF2RB genes across two survival indices. *OS* Overall Survival, *PFS* Progression-Free Survival

The heatmap distinctly delineates the impact of various genes on survival metrics, notably demonstrating that the KCNN4 gene exhibits elevated hazard ratios (HR values of 1.16, and 1.43) across all two survival indices: overall survival (OS), and progression-free survival (PFS) (Fig. 9). This correlation suggests that high expression of KCNN4 is closely associated with poorer prognoses in patients. Such findings underscore the significance of KCNN4 as a potential biomarker for tumor progression, highlighting its pivotal role in oncological research and clinical assessments. To further investigate the biological role of KCNN4, we transfected PC9 cells with the silencing plasmid sh-KCNN4 and control shNC, conducting subsequent studies.

### Experimental validation

#### Gene expression of KCNN4 is downregulated by ShRNA

**Fig. 10:**
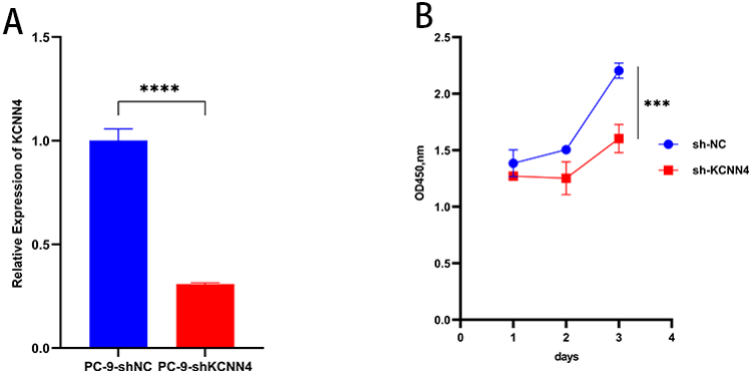
KCNN4 Knockdown Reduces Expression and Cell Proliferation in PC-9 Cells. **A** Relative mRNA expression of KCNN4 in PC-9 cells transfected with shKCNN4 versus non-targeting control shRNA (shNC). Expression normalized to GAPDH. Mean ± SEM; **B** Proliferation of PC-9 cells after KCNN4 knockdown, measured over three days using the CCK-8 assay. Mean ± SEM; ***p < 0.001., ****p < 0.0001. All experiments were repeated three times

To investigate the role of KCNN4 in PC-9 cell function, we first examined the relative expression of KCNN4 using RNA interference. Following the transfection of PC-9 cells with shRNA specific to KCNN4 (PC-9-shKCNN4) and a non-targeting control shRNA (PC-9-shNC), real-time quantitative PCR was conducted. The viral transfection of PC-9 cells was optimized to achieve high transfection efficiency, as evidenced by the robust expression of the EGFP reporter gene. Images were analyzed in ImageJ/Fiji. EGFP-positive cells were identified using a fixed intensity threshold and object-size criteria, and the EGFP-positive fraction (%) was calculated per field. The resulting efficiencies were shNC: 96.33 ± 0.88% and shKCNN4: 95.67 ± 0.59%, with no significant difference between groups.(*p* = 0.34). Representative EGFP fluorescence micrographs of the final transduction outcome are shown in Supplementary Figure *SEGFP.* KCNN4 expression in the shKCNN4 group was significantly reduced compared to the control group (Fig. 10A). Specifically, the expression of KCNN4 in PC-9-shKCNN4 cells was approximately 75% lower than in PC-9-shNC cells, indicating effective knockdown by shRNA (****p < 0.0001).

#### Knockdown of KCNN4 reduces cell proliferation

Using the CCK-8 assay, two-way repeated-measures ANOVA with Geisser–Greenhouse correction showed significant main effects of group and time and a group×time interaction, indicating that the anti-proliferative effect of KCNN4 silencing intensifies over time (group: F(1, 8) = 96.80, *P* < 0.0001; time: F(1.87, 14.98) = 99.16, *P* < 0.0001; interaction: F(2, 16) = 15.43, *P* = 0.0002).Post-hoc Šídák comparisons identified the earliest significant separation at day 2 (sh-NC 1.50 ± 0.04 vs. sh-KCNN4 1.25 ± 0.15; mean difference 0.25 [95% CI 0.00–0.50]; adjusted *P* = 0.05). By day 3, the deficit was large and highly significant (sh-NC 2.21 ± 0.07 vs. sh-KCNN4 1.60 ± 0.13; mean difference 0.60 [95% CI 0.40–0.81]; adjusted *P* = 0.0002).These findings demonstrate a time-dependent and quantitatively large suppression of PC-9 proliferation following KCNN4 knockdown.

#### Silencing KCNN4 inhibited the migration and invasion of PC9 cells

**Fig. 11:**
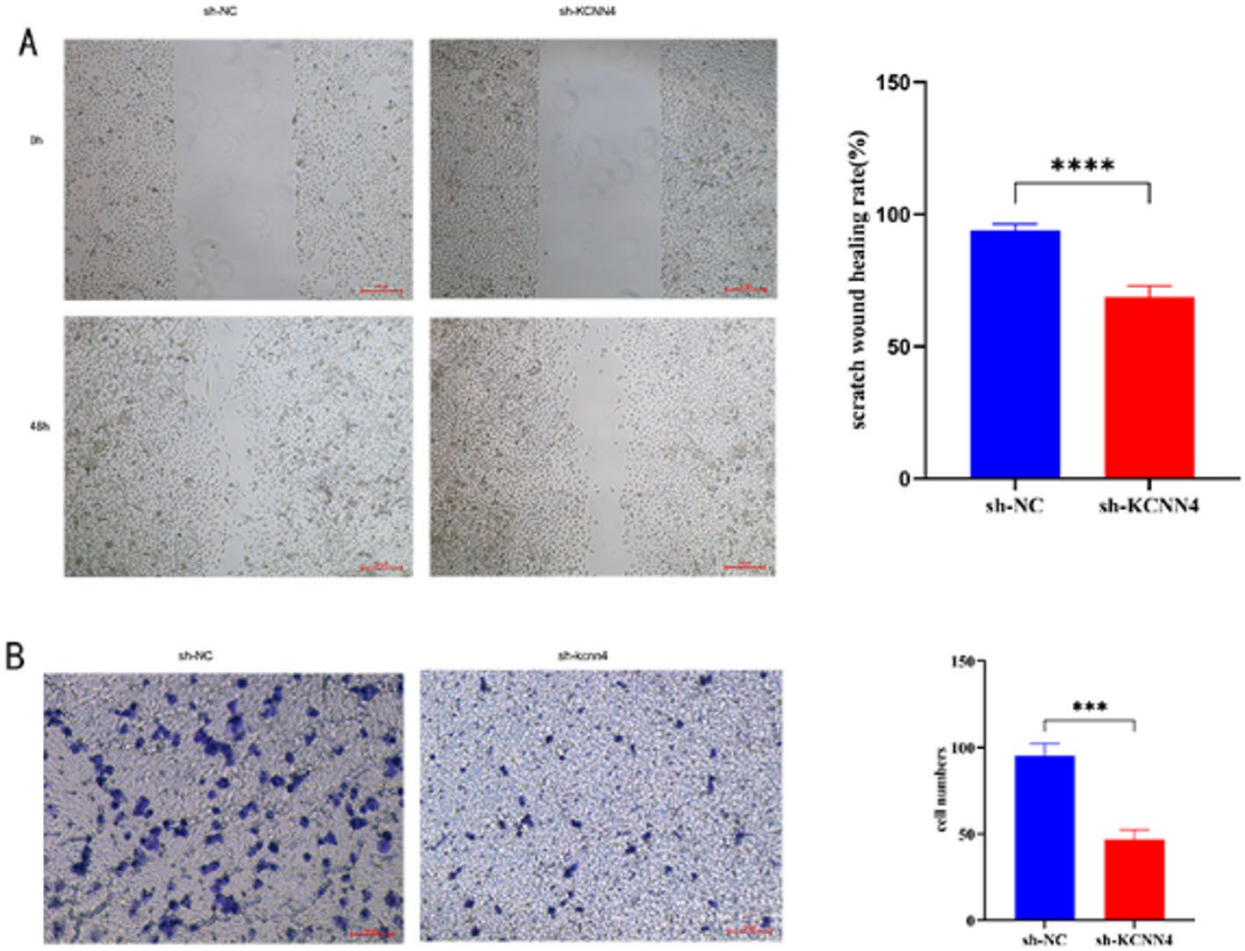
Impact of KCNN4 Knockdown on PC9 Lung Cancer Cell Migration and Invasion. **A** Scratch assay comparison of cell migration in PC9 cells transfected with sh-NC (control) versus sh-KCNN4, showing significantly reduced migration in the sh-KCNN4 group at 48 h post-scratch. **B** Transwell invasion assay results demonstrating a marked decrease in the invasion capability of PC9 cells with KCNN4 knockdown compared to controls, with statistical significance (*P* < 0.001). ****p < 0.0001, ***p < 0.001. All experiments were repeated three times

Knocking down KCNN4 also inhibited the migration and invasion of lung adenocarcinoma cells. Migration is a critical characteristic of lung cancer cells. We studied the impact of KCNN4 gene knockdown on the migration ability of PC9 cells using a scratch assay. The results showed a significant decrease in the healing rate for the sh-KCNN4 group compared to the sh-NC group (93.95% ± 2.34 vs. 68.71% ± 4.12, *P* < 0.0001).( Fig. 11A) Furthermore, we explored the effect of KCNN4 knockdown on the invasive capabilities of PC9 cells through a Transwell assay. The number of cells that migrated through the membrane was found to be significantly lower in the sh-KCNN4 group compared to the sh-NC group (46.75 ± 6.66 vs. 95.33 ± 7.02, *P* < 0.0001) (Fig. 11B).

#### Molecular Docking and molecular dynamics simulations

**Fig. 12:**
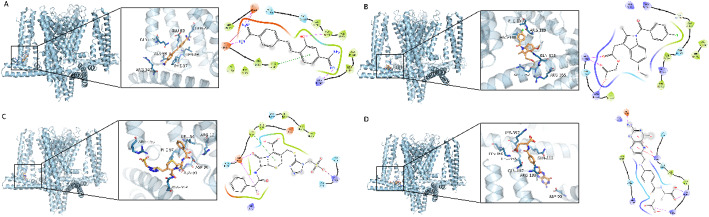
Molecular Docking Analysis of KCNN4 Protein with Selected FDA-Approved Small Molecules. **A** The 3D and 2D interaction diagrams of the molecular docking of ZINC000000001547 (Hydroxystilbamidine) with KCNN4 protein. The 3D view shows the small molecule (orange sticks) bound to the protein (blue cartoon). The 2D interaction map indicates hydrogen bonds with residues GLU-83, GLU-22, ALA-99, ARG-342, pi-pi conjugation with PHE-87, and salt bridges with GLU-22 and GLU-83. **B** The docking interactions of ZINC000000601272 (Acemetacin) with KCNN4 protein are illustrated. The 3D model depicts the binding of the molecule to the protein’s active site. The 2D interaction details highlight hydrogen bonds with ARG-352 and salt bridges with the same residue. **C** Visualization of ZINC000003830428 (Cefonicid) interactions with KCNN4. The 3D image shows binding at the helix crossing of the protein, while the 2D map reveals hydrogen bonds with ASN-91 and ASP-90, pi-pi interactions with PHE-87, and salt bridges with ARG-103. **D** Depiction of ZINC000008577218 (Pga) docking with KCNN4. The 3D structure indicates binding within the protein’s active site. The 2D interaction map shows hydrogen bonds with LYS-357 and ASP-90, pi-pi conjugation with ARG-352, and salt bridges with LYS-360 and LYS-357

The virtual screening of 3,330 FDA-approved small molecules from the ZINC database resulted in 3,013 successfully docked compounds. The top 20 compounds demonstrated docking scores ranging from − 8.153 to -9.001 kcal/mol(Table S6), with the highest affinity compounds being ZINC000000001547 (Hydroxystilbamidine), ZINC000000601272 (Acemetacin), ZINC000008577218 (Pga), and ZINC000003830428 (Cefonicid).( Fig. 12) Detailed binding mode analyses revealed that these compounds form multiple hydrogen bonds, π-π stacking interactions, and salt bridges with key residues of the KCNN4 protein, indicating their strong binding affinities.

**Fig. 13:**
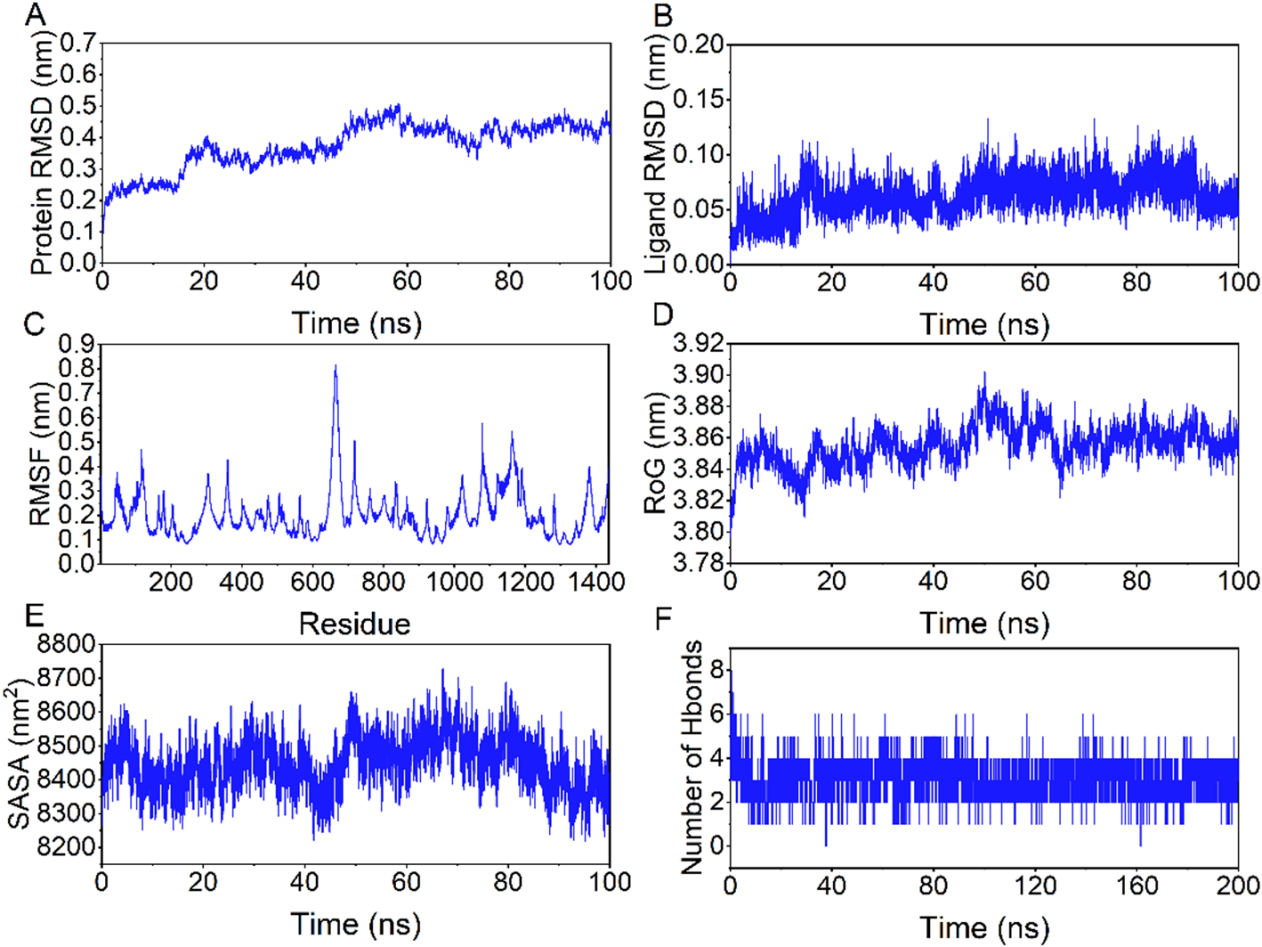
Molecular Dynamics Simulations of KCNN4 Protein with ZINC000000001547 (Hydroxystilbamidine). **A** Protein Backbone RMSD - The Root Mean Square Deviation (RMSD) of the protein backbone atoms over time indicates system stability post-50 ns, maintaining around 0.45 nm, signifying a stable protein structure conducive for ligand binding. **B** Ligand RMSD - The RMSD of the ligand shows consistent binding within the protein’s active site, with fluctuations remaining within 0.15 nm throughout the simulation, indicating stable interaction within the binding pocket. **C** Protein RMSF - The Root Mean Square Fluctuation (RMSF) of the protein residues reveals most residues fluctuating within 0.3 nm, indicating low flexibility and suggesting a stable protein conformation. **D** Radius of Gyration (RoG) - The Radius of Gyration shows the compactness of the protein-ligand complex, with values stabilizing after 70 ns, indicating a tightly bound and stable complex. **E** Solvent Accessible Surface Area (SASA) - The Solvent Accessible Surface Area (SASA) of the complex remains consistent, indicating stable exposure to the solvent and suggesting a steady-state conformation in the aqueous environment. **F** Hydrogen Bonds - The number of hydrogen bonds between the protein and the ligand fluctuates around 3, demonstrating significant interactions that contribute to the stability of the protein-ligand complex

Among the 4 × 20 molecular docking results, four molecules—ZINC000000001547 (Hydroxystilbamidine), ZINC000000601272 (Acemetacin), ZINC000003830428 (Cefonicid), and ZINC000008577218 (Pga)—demonstrated significant binding affinity with the KCNN4 protein. We selected the best-binding drug small molecule with KCNN4 for further interaction analysis. The molecule showing the most satisfactory binding was ZINC000000001547 (Hydroxystilbamidine) with KCNN4, which was then subjected to molecular dynamics (MD) simulation to further validate the reliability of the molecular docking results.The molecular dynamics (MD) simulations of the KCNN4 protein with ZINC000000001547 (Hydroxystilbamidine) demonstrated stable interactions and structural integrity .Over the 0–100 ns trajectory, the protein backbone RMSD averaged 0.37 ± 0.074 nm, and within the 50–100 ns stable window it was 0.430 ± 0.028 nm (stable time 50.01%), consistent with equilibration near 0.43–0.45 nm (Fig. 13A). The complex RMSD showed a similar pattern (0.419 ± 0.074 nm overall; 0.476 ± 0.028 nm during 50–100 ns; stable time 50.01%), indicating a globally stable complex. The ligand RMSD remained low throughout (0.063 ± 0.017 nm overall and 0.063 ± 0.017 nm in the 0–100 ns stable window; stable time 100.00%) (Fig. 13B), reflecting persistent confinement within the binding pocket. Per-residue flexibility was modest, with RMSF (median [IQR]) = 0.183 nm [0.143–0.239 nm] (Fig. 13C). The radius of gyration (Rg) was 3.855 ± 0.013 nm overall and 3.859 ± 0.008 nm in the 70–100 ns stable window (stable time 30.01%), consistent with a compact protein–ligand assembly (Fig. 13D). The solvent-accessible surface area (SASA) remained nearly constant (844.8 ± 7.5 nm² overall and in the 0–100 ns stable window; stable time 100.00%) (Fig. 13E). Protein–ligand hydrogen bonding averaged 3.06 ± 0.73 over the trajectory; among frames with ≥ 3 concurrent hydrogen bonds, the mean count was 3.31 ± 0.54, and the complex maintained ≥ 3 hydrogen bonds for 81.93% of simulation frames (Fig. 13F). Collectively, these quantitative metrics substantiate the visual trends and support a stable, well-packed KCNN4–Hydroxystilbamidine complex throughout the MD simulation. These findings validate the reliability of the molecular docking results, confirming ZINC000000001547 as a promising candidate for further drug development targeting KCNN4.

## Discussion

This study aimed to elucidate the expression patterns of ICD-related genes in NSCLC and their prognostic significance and role in the tumor microenvironment (TME). We identified three distinct lung cancer subtypes based on ICD-related gene expression, each with unique immune microenvironment characteristics and prognostic implications.

Gene Ontology (GO) analysis reveals that ICD-related differentially expressed genes (DEGs) are predominantly enriched in biological processes related to immune responses, inflammatory processes, and adaptive immunity. This suggests that these genes play a pivotal role in modulating the immune microenvironment and inflammatory responses in lung cancer. Genes such as IL6 and CALR are central to immunogenic cell death. According to research by Asma Ahmed and S. Tait, IL6, as one of the damage-associated molecular patterns (DAMPs), facilitates the activation of tumor-specific immune responses during the ICD process. These DAMPs enhance the long-term efficacy of anticancer drugs by inducing immune system attacks on tumors, where IL6 promotes inflammatory and immune responses, indirectly influencing the process and outcome of ICD [20]. Calreticulin (CALR), an intracellular molecule, relocates to the cell surface during cell death, serving as an “eat me” signal that prompts antigen-presenting cells, such as dendritic cells, to recognize and phagocytize dying tumor cells. This process triggers an immune response against tumor cells, with studies by Kielbik et al. highlighting CALR’s potential role as a biomarker in the prognosis of ovarian cancer patients, underscoring its critical function in ICD [21]. Th17 cell differentiation promotes immune evasion in NSCLC, facilitating tumor progression and offering a potential avenue for therapeutic intervention [22].

Our unsupervised clustering analysis identified three distinct subtypes of NSCLC based on their unique immune profiles and prognoses. Cluster 1, distinguished by high immune cell infiltration, is associated with a better survival prognosis, suggesting that patients within this cluster may derive significant benefit from immunotherapy. This observation aligns with existing literature indicating that elevated levels of tumor-infiltrating lymphocytes (TILs), particularly CD8^+^ T cells, are linked to improved outcomes in lung cancer [16, 17]. Patients within Cluster 1 may be ideal candidates for immune checkpoint inhibitors (ICIs), such as anti-PD-1 or anti-PD-L1 therapies, which have demonstrated significant efficacy in patients with an inflamed tumor microenvironment (TME). The high immune infiltration suggests that these patients may experience more durable responses to ICIs compared to other subtypes. In clinical practice, the identification of Cluster 1 patients can guide the early adoption of ICIs, potentially in combination with other immune-activating agents, to maximize therapeutic benefits. Additionally, regular assessment of TIL levels could be implemented as a biomarker for treatment monitoring, providing dynamic insight into the patient’s response to immunotherapy. Conversely, Cluster 2 is characterized by a reduction in effector memory T cells, indicative of an immune desert phenotype associated with poor prognosis. This phenotype underscores the necessity for alternative therapeutic strategies, as patients in this cluster are likely to exhibit resistance to conventional immunotherapies [23, 24]. Instead, combination strategies designed to re-engage the immune system, such as the co-administration of ICIs with chemotherapy or targeted therapies, may be more effective.Finally, Cluster 3 is characterized by increased tumor purity and lower immune scores, suggesting a more aggressive tumor phenotype that may require more intensive treatment regimens. High tumor purity correlates with diminished immune infiltration, often resulting in poorer clinical outcomes. Given the high tumor burden and low immune engagement in Cluster 3, patients may require more aggressive multimodal therapies, including chemotherapy, targeted therapy, or radiation, to achieve disease control. Furthermore, genomic profiling may reveal actionable mutations (e.g., KRAS, EGFR, or ALK) that could be targeted with specific inhibitors, offering another avenue for personalized treatment [25]. These findings provide critical insights into the immune landscapes and prognoses of NSCLC subtypes, guiding tailored therapeutic strategies to improve patient outcomes. Clinical evidence supports our subtype-based therapeutic suggestions. For example, patients with high immune infiltration show significantly better responses to immune checkpoint inhibitors (ICIs), as demonstrated in large clinical trials [26]. These findings align with our classification, suggesting that the immune-inflamed subtype may derive the greatest benefit from immunotherapy.

Among the five signature genes, KCNN4 emerged as a critical prognostic marker [27].and therapeutic target Recent advances in cancer research have implicated KCNN4, a potassium calcium-activated channel, as a critical player in the TME, influencing both cancer progression and responses to therapy across various cancer types. For example, Cytoplasmic ACYP2 negatively regulates the expression of KCNN4, thereby suppressing K⁺ efflux, leading to inactivation of the ERK signaling pathway and, consequently, impeding hepatocellular carcinoma (HCC) growth and metastasis [28]. KCNN4 is recognized as a potential prognostic biomarker owing to its critical role in modulating the tumor microenvironment (TME). Chen et al. [29] emphasized that KCNN4 expression is abnormal in various cancers, correlating with tumor mutational burden (TMB), microsatellite instability (MSI), and immune checkpoint genes (ICGs). This correlation suggests its potential utility in predicting responses to immunotherapy.In papillary thyroid cancer (PTC), KCNN4 has been identified to facilitate cancer progression by promoting epithelial-mesenchymal transition and inhibiting apoptosis, indicating its role in sustaining aggressive cancer phenotypes [30]. This oncogenic role was similarly observed in kidney renal clear cell carcinoma, where KCNN4 expression was associated with poorer prognoses and its modulation affected immune cell infiltration in the TME [31] .Further supporting its role in metabolic pathways, KCNN4 was shown to enhance the glucose metabolism in liver cancer stem cells (LCSCs), promoting their stemness and resistance to therapies, which provides a potential target for novel therapeutic strategies aimed at eradicating LCSC [32]. Moreover, KCNN4 may intersect with ICD signaling pathways. For instance, abnormal KCNN4 activity could influence the exposure of calreticulin (CALR) or the extracellular release of HMGB1, both of which are key DAMPs that activate antigen-presenting cells and initiate antitumor immune responses [33]. This suggests that KCNN4 not only regulates tumor progression through metabolic reprogramming but may also shape the tumor immune microenvironment by modulating ICD-related signaling.

Beyond oxidative phosphorylation, it is important to consider the role of KCNN4 in broader cancer-metabolic pathways. The Warburg effect, a hallmark of cancer metabolism, describes the preference of tumor cells for aerobic glycolysis even under normoxic conditions, thereby supporting rapid ATP production and macromolecule synthesis to sustain proliferation. Recent evidence suggests that KCNN4 may also contribute to glycolytic regulation. For instance, Fan et al. reported that KCNN4 enhances glucose metabolism and maintains stemness properties in liver cancer stem cells, which indicates a potential link between KCNN4 activity and glycolytic flux [32]. These findings imply that KCNN4 may act as a key modulator of the metabolic balance between oxidative phosphorylation and glycolysis. A deeper comparative exploration of these alternative metabolic routes will not only clarify the mechanistic role of KCNN4 in metabolic reprogramming but may also uncover novel therapeutic strategies targeting cancer metabolism.

Among the 5 signature-associated genes(CSF2RB、CD3D、ADA2、KCNN4、AREG), Recent studies have shed light on the complex roles of Colony Stimulating Factor 2 Receptor Beta (CSF2RB) in various cancers, suggesting both prognostic and therapeutic implications. CSF2RB, a critical component of cytokine receptor complexes, has been implicated in oncogenic transformations and immune regulation within the tumor microenvironment (TME). In breast cancer, Rashid et al. [34] discovered a novel somatic mutation in CSF2RB, which appears to have transformative properties, highlighting its potential role in oncogenesis through ligand-independent signaling pathways. This mutation facilitates survival and proliferation under ligand starvation, primarily through the JAK2/STAT and PI3K/mTOR pathways, highlighting a possible target for therapeutic intervention. Moreover, in lung adenocarcinoma, CSF2RB expression correlates with immune infiltration, particularly affecting macrophage dynamics and tumor progression. Zhu et al. [35] demonstrated that CSF2RB serves as a unique biomarker linked with early stages of lung cancer and is inversely related to patient survival, making it a potential target for enhancing immune-based therapies. The role of CSF2RB in promoting immune evasion was also noted in brain metastases from breast cancer. Klemm et al. [36] reported that CSF1R inhibition leads to compensatory activation of CSF2RB, which mitigates the efficacy of CSF1R-targeted therapies. This compensatory mechanism via CSF2RB supports the tumor microenvironment by enhancing macrophage-mediated inflammatory responses, suggesting that dual inhibition of CSF1R and CSF2RB could be more effective.

CD3D has emerged as a significant prognostic marker across various cancers. Notably, in colon cancer, its expression shows an inverse correlation with clinical staging, while positively influencing patient survival outcomes. Studies like those by Yang et al. [37] have demonstrated that higher CD3D expression is associated with better clinical outcomes in colon adenocarcinoma, making it a potential marker for prognosis and a target for immunotherapy strategies In head and neck squamous cell carcinoma (HNSCC), CD3D expression has been linked to enhanced immune infiltration and an improved response to immunotherapeutic agents. Research by Wei et al. found that high CD3D levels were significantly associated with longer overall survival, suggesting its role as an independent favorable prognostic biomarker [38].

ADA2, primarily known for its role in purine metabolism by converting adenosine into inosine, has been shown to exhibit distinct roles in various cancers. For instance, Gao et al. [39] provided a comprehensive analysis of ADA1 and ADA2 across multiple cancer types, revealing that ADA2 is significantly increased in certain cancers like esophageal carcinoma, glioblastoma multiforme, and pancreatic adenocarcinoma. Importantly, they illustrated that elevated ADA2 expression correlates with a favorable prognosis in multiple cancers, including those of the breast, cervix, head and neck, and lungs. Amphiregulin (AREG), belonging to the epidermal growth factor (EGF) family, has been identified as a pivotal factor in the pathology of numerous cancers. This molecule exerts its effects on tumor progression primarily through its interaction with the epidermal growth factor receptor (EGFR), thereby playing a crucial role in cancer development and progression. Recent research highlights AREG’s multifaceted roles, including its impact on the EMT, resistance to chemotherapy, and the tumor microenvironment.

AREG has been shown to facilitate EMT in pancreatic cancer cells through the activation of the EGFR/ERK/NF-κB signaling pathway. The study by Wang et al. [40] elucidated how AREG facilitates the metastatic potential of pancreatic cancer cells by enhancing their migratory and invasive capabilities. This effect is mediated through increased nuclear accumulation of NF-κB, which is triggered by the EGFR/ERK pathway upon AREG stimulation, leading to changes in the expression of key EMT markers such as E-cadherin, vimentin, Snail, and Slug.

The model effectively distinguishes between high-risk and low-risk patients, with high-risk individuals demonstrating worse overall survival. This stratification enables clinicians to tailor treatment intensity according to the patient’s risk profile. For instance, low-risk patients, with better prognoses, may benefit from less aggressive treatment modalities or may be candidates for immunotherapy as a first-line option, given their likely favorable response to such interventions. Conversely, high-risk patients, characterized by elevated expression of genes like KCNN4 and AREG, may require more aggressive and targeted therapies to improve their survival prospects. These patients may also be prime candidates for clinical trials involving novel therapeutic agents or combination treatments aimed at overcoming resistance mechanisms.

Additionally, we further applied molecular docking and molecular dynamics simulations to screen four of the most promising small-molecule drugs (ZINC000000001547 (Hydroxystilbamidine), ZINC000000601272 (Acemetacin), ZINC000008577218 (Pga), and ZINC000003830428 (Cefonicid)). These findings provide novel targets and potential drugs for the development of new therapeutics for lung cancer.

The limitations of this study encompass the relatively small sample size and the dependence on in vitro validation. The limited sample size may compromise the statistical power of subgroup analyses, potentially leading to unstable or non-generalizable results. First, in vivo validation of ICD-related subtypes is critical to confirming their clinical relevance. Expanding the validation cohort using additional GEO datasets could improve the robustness of our findings, while animal models of NSCLC that replicate the immune profiles of the identified subtypes would allow more reliable evaluation of therapeutic responses. Animal models of NSCLC that replicate the immune profiles of the identified subtypes could be developed to assess their responses to therapies, particularly immune checkpoint inhibitors (ICIs). The knockdown of key genes like KCNN4 in these models could provide additional insights into their role in tumor progression and immune evasion. Moreover, KCNN4 may regulate ICD signaling through pathways such as CALR exposure and HMGB1 release, thereby influencing antigen presentation and immune activation; this mechanistic link requires further experimental confirmation Second, therapeutic development and testing: The small-molecule drugs identified through molecular docking, such as ZINC000000001547 (Hydroxystilbamidine), could be tested in vivo for their efficacy in inhibiting KCNN4 and reducing tumor aggressiveness. Optimization of these compounds to improve potency and bioavailability could further support their development as therapeutic agents. In addition, developing biomarker panels based on the immune signatures of the identified subtypes (e.g., activated CD4 + and CD8 + T cells for ICI responsiveness) could aid in predicting patient responses to immunotherapies. These biomarker panels could be incorporated into clinical trials to stratify patients and refine treatment selection.

## Conclusion

This study identifies three distinct ICD-related TIME subtypes in NSCLC with significant translational potential: Cluster 1 patients are ideal candidates for immune checkpoint inhibitors; Cluster 2 may benefit from combination therapies to overcome resistance; and Cluster 3 requires aggressive multimodal treatments. Additionally, the predictive model enhances risk stratification, improving patient outcomes in NSCLC.

## Electronic Supplementary Material

Below is the link to the electronic supplementary material.

Supplementary Material 1: Table S1

Supplementary Material 2: Table S2

Supplementary Material 3: Table S3

Supplementary Material 4: Table S4

Supplementary Material 5: Table S5

Supplementary Material 6: Table S6

## Data Availability

The datasets produced and/or utilized in this study can be obtained from the corresponding author upon reasonable request.
